# Jiedu Huoxue decoction inhibits cardiomyocyte apoptosis via PTEN/AKT/GSK3β-mediated mitochondrial dynamics in myocardial infarction: an integrative study of network pharmacology, transcriptomics and molecular docking

**DOI:** 10.1186/s13020-026-01444-7

**Published:** 2026-06-29

**Authors:** Mingjie Pang, Han Peng, Changlei Hu, Yutong Li, Yangzhen Pei, Bin Liu, Yuting Wu, Guanghong Chen, Haozhen Cui, Aihua Shen, Li Zheng, Lingpeng Xie, Yingchun Zhou, Guoyong Zhang, Xin Han

**Affiliations:** 1 Department of Traditional Chinese Medicine, Medical College, Yanbian University, Yanji, Jilin China; 2 School of Traditional Chinese Medicine, Southern Medical University, Guangzhou, China; 3 The First Affiliated Hospital of Guangzhou University of Chinese Medicine, Guangzhou, China; 4 Department of Traditional Chinese Medicine, The Second Affiliated Hospital of Guangzhou Medical University, Guangzhou Medical University, Guangzhou, China; 5 Binzhou Medical University Hospital, Binzhou, 256603 China; 6 Department of Cardiovascular Medicine, The First Affiliated Hospital of Guangzhou University of Chinese Medicine, Guangzhou, Guangdong China; 7 The Affiliated Traditional Chinese Medicine Hospital, Guangzhou Medical University, Guangzhou, China

**Keywords:** JDHXD, Myocardial infarction, Mitochondrial dynamics, Cardiomyocyte apoptosis, PTEN/AKT/GSK3β

## Abstract

**Background:**

Myocardial infarction (MI) triggers oxidative stress, mitochondrial dysfunction, and cardiomyocyte apoptosis. At present, it remains urgently needed to develop novel therapies specifically suppressing cardiomyocyte apoptosis via improving mitochondrial dysfunction following MI. Jiedu Huoxue Decoction (JDHXD) may have the effect of ameliorating myocardial injury after MI.

**Purpose:**

This study examined the protection exerted by JDHXD against myocardial injury post-MI and investigated the underlying mechanisms of action.

**Methods:**

UHPLC/Orbitrap-MS, network pharmacology and transcriptome analysis were used to study the effective components and potential targets of JDHXD for treating MI. In vivo: The MI mice received JDHXD (12.74/25.48 g/kg/day) or captopril treatment for 28 days. Later, cardiac function (tested by echocardiography and histopathology), apoptosis, oxidative stress, mitochondrial ultrastructure, mitochondrial fission/fusion and PTEN/AKT/GSK3β pathway protein levels were evaluated. In vitro: TBHP-induced cardiomyocytes (H9C2 cells and NCMs) were exposed to JDHXD treatment (50–200 μg/mL), with or without PTEN inhibitor Bpv (2 μM) or PTEN overexpression (through adenoviral transduction). Afterwards, cell apoptosis, oxidative stress, mitochondrial membrane potential, and relative proteins were assayed. Experiments such as molecular docking and surface plasmon resonance imaging (SPRi) were conducted to verify the effective components of JDHXD in preventing myocardial mitochondrial injury after MI.

**Results:**

The results of network pharmacology and transcriptomics suggest that JDHXD may ameliorate myocardial injury after MI through modulating PTEN for activating the PI3K/AKT/GSK3β signaling pathway. In vivo: JDHXD dose-dependently improved left ventricular function, improved the oxidative stress-induced imbalance of mitochondrial fission/fusion, and inhibited cardiomyocyte apoptosis post-MI in association with suppressing the PTEN/AKT/GSK3β pathway. In vitro: JDHXD suppressed the TBHP-induced cardiomyocyte apoptosis, attenuated oxidative stress, preserved mitochondrial potential, and restored mitochondrial dynamics. PTEN inhibitor did not augment JDHXD’s effects, whereas PTEN overexpression partially abolished JDHXD’s protection against myocardial injury induced by oxidative stress. UHPLC/Orbitrap-MS, molecular docking, SPRi and experiments in vitro confirmed that puerarin is one of the main components of JDHXD in regulating the PTEN/AKT/GSK3β pathway to improve mitochondrial function after MI and inhibit cardiomyocyte apoptosis.

**Conclusions:**

JDHXD against oxidative stress-induced cardiomyocyte apoptosis post-MI through ameliorating mitochondrial dysfunction, which is partially mediated by suppressing the PTEN/AKT/GSK3β pathway to inhibit excessive mitochondrial fission and promote mitochondrial fusion.

**Graphical Abstract:**

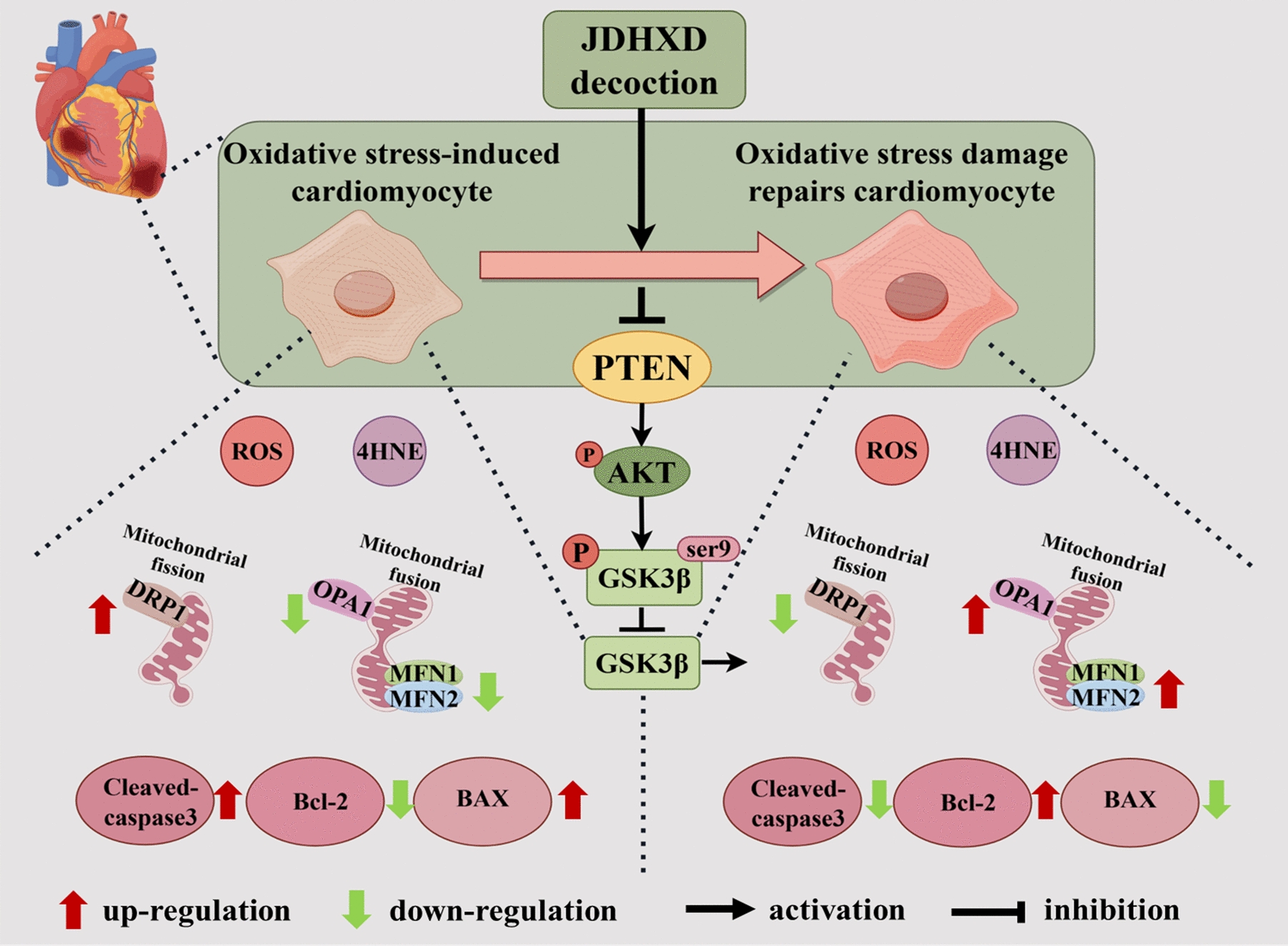

**Supplementary Information:**

The online version contains supplementary material available at 10.1186/s13020-026-01444-7.

## Introduction

As the cardiovascular condition, myocardial infarction (MI) is marked by left ventricular structural and functional abnormalities, which remains a major factor inducing death globally. Its annual incidence and mortality rates show a continuing upward trend [1, 2]. Although reperfusion therapy and guideline-directed medical therapy (ACE inhibitors, ARBs, MRAs, and beta-blockers) can dramatically decrease the death rate of MI patients, it is still associated with a high residual subsequent heart failure (HF) risk [3, 4]. Therefore, elucidating the underlying mechanisms of MI progression and identifying novel anti-MI treatments remain imperative.

MI represents the complex pathology that encompasses inflammation, cardiomyocyte apoptosis, cardiac fibrosis, oxidative stress, as well as mitochondrial energetic dysfunction. Collectively, these mechanisms drive MI progression [5–7]. Its detrimental outcomes can be predominantly driven by progressive death of cardiomyocytes, in particular apoptosis [8, 9]. Thus, suppressing apoptosis in cardiomyocytes is an important intervention to ameliorate post-MI myocardial injury [10]. Mounting evidence has emphasized that mitochondrial bioenergetics is important for MI, whose disruption in both biogenesis and function serves as a hallmark feature of ensuing cardiac injury. Accounting for 20%–35% of cardiomyocyte volume, mitochondria constitute the principal energy source for cardiac muscle. Homeostatic maintenance of mitochondrial structure and function relies critically on the balanced fission/fusion dynamics [11]. Sustained hypoperfusion and hypoxia in the infarcted myocardium can provoke pathological accumulation of reactive oxygen species (ROS), driving aberrant mitochondrial fission and opening of mitochondrial permeability transition pores (mPTPs) [12, 13], while its persistent activation induces profound mitochondrial dysfunction marked by membrane depolarization. Ultimately, it intensifies cytochrome c (Cytc) release by cells and initiates the Bcl-2/BAX axis as well as the downstream pro-apoptotic caspase pathway, inducing apoptosis [7, 14–16]. Collectively, the above results indicate that improving myocardial mitochondrial injury caused by oxidative stress is an effective way to protect cardiac function and inhibit cardiomyocyte apoptosis after MI.

Phosphatase and tensin homolog (PTEN), a lipid/protein phosphatase, regulates cellular homeostasis to modulate cell growth and survival, and its functions primarily manifests as tumor suppression [17]. PTEN can dephosphorylate phosphatidylinositol (3,4,5)-triphosphate (PIP3), while PTEN inhibition can significantly up-regulate the PI3K/AKT pathway [18]. While PTEN drives oncogenesis in cancer syndromes, our previous studies reveal that PTEN suppression post-MI can significantly promote the phosphorylation level of AKT to confer myocardial protection [19, 20]. Furthermore, in some research, cytosolic Akt activation alone cannot confer organ protection until its mitochondrial translocation and target protein phosphorylation [21, 22]. Two mitochondrial proteins, namely, ATP synthase β-subunit and glycogen synthase kinase 3β (GSK-3β), are the phosphorylation substrates following mitochondrial Akt activation [23]. Of them, GSK-3β acts as a multifunctional kinase that critically regulates mitochondrial dynamics [24], and participates in fission/fusion processes by phosphorylating Drp1 and OPA1 to alleviate oxidative stress-related apoptosis of cardiomyocytes [25–27]. Consequently, regulating GSK-3β through PTEN to rescue cardiomyocyte apoptosis caused by abnormal mitochondrial dynamics is the efficient way for repairing myocardial injury post-MI.

Our previous research confirms that Taohong Siwu Decoction (THSWD), a classic TCM formula with blood circulation-promoting effects, significantly inhibits cardiomyocyte apoptosis post-MI [20]. Jiedu Huoxue Decoction (JDHXD) is a herbal formula derived from THSWD, which was originally recorded in Yilin Gaicuo (Corrections on the Errors of Medical Works) by Wang Qingren in the Qing Dynasty to promote detoxification and blood circulation in treating cardiovascular diseases. JDHXD consists of Puerariae Radix, Paeoniae Radix Rubra, Persicae Semen, Angelicae Sinensis Radix, Rehmanniae Radix, Carthami Flos, Forsythiae Fructus, Bupleuri Radix, Aurantii Fructus Immaturus and Glycyrrhizae Radix et Rhizoma. Clinical studies have confirmed that JDHXD can significantly improve the cardiac function indicators after MI [28]. Previous in vivo pharmacological studies have demonstrated that JDHXD exerts protective effects in experimental models, including alleviating myocardial ischemia–reperfusion injury by regulating inflammatory responses, promoting autophagy and weakening ventricular remodeling [29, 30], which is consistent with its traditional application for conditions involving blood stasis and toxin accumulation [31]. Nonetheless, it remains unclear whether JDHXD can improve myocardial injury by alleviating mitochondrial dysfunction post-MI.

This study combined in vivo murine MI models with in vitro tert-butyl hydroperoxide (TBHP)-induced cardiomyocyte injury experiments to investigate whether JDHXD and its active ingredients protected against oxidative stress-induced myocardial mitochondrial injury to suppress cardiomyocyte apoptosis during MI. Besides, in vitro experimental validations were carried out with specific inhibitors and adenoviruses for investigating the anti-MI mechanism of JDHXD.

## Materials and methods

### Materials

JDHXD is composed of multiple herbal components (Table 1). The herbal medicines were provided by the Affiliated Hospital of Yanbian University (Yanji, Jilin, China). Puerarin (purity ≥ 98%) was acquired in Mansite Biotechnology (Chengdu, China). JC-1 mitochondrial membrane potential (MMP) fluorescent probe and ROS assay kit were purchased in Solarbio (Beijing, China). Anti-PTEN (AF6351), anti-p-PTEN (AF4450), anti-p-AKT (AF0016), anti-p-GSK3β(Ser9) (AF2016), anti-BAX (AF0120), anti-GSK3β (AF5016), anti-Bcl-2 (AF6139), anti-Caspase3 (AF6311), anti-Cleaved-Caspase3 (Asp175), anti-p-DRP1 (AF8470), and anti-vimentin (BF8006) antibodies were obtained from Affinity Biosciences (Ohio, USA). Anti-4-HNE antibody (ab46545) was acquired in Abcam (Cambridge, MA, USA), anti-cardiac troponin T (cTnT) antibody (BS6013) in Bioworld Technology (Atlanta, GA, USA), and anti-GAPDH antibody in Cell Signaling Technology (Beverly, MA, USA). Anti-MFN1 (13798-1-AP), anti-OPA1 (27733-1-AP), and anti-MFN2 (12186-1-AP) antibodies were obtained in Proteintech (Chicago, IL, USA). Bpv (HOpic) (#S8651) was obtained from Selleck Chemicals (USA). H9C2 cells were procured in the Shanghai Institutes for Biological Sciences, whereas Annexin V-FITC/PI Apoptosis Detection Kit in BD Biosciences (USA). Other chemical reagents were supplied by respective commercial providers.

**Table 1:** The drug information in JDHXD

Chinese name	Botanical name	Dosage used	Full botanical names
Di Huang	Rehmanniae Radix	15 g	*Rehmannia glutinosa* (Gaertn.) Libosch. ex DC
Tao Ren	Persicae Semen	25 g	*Prunus persica* (L.) Batsch
Hong Hua	Carthami Flos	15 g	*Carthamus tinctorius* L
Chi Shao	Paeoniae Radix Rubra	10 g	*Paeonia lactiflora* Pall
Ge Gen	Puerariae Radix	5 g	*Pueraria lobata* (Willd.) Ohwi
Lian Qiao	Forsythiae Fructus	5 g	*Forsythia suspensa* (Thunb.) Vahl
Zhi Qiao	Aurantii Fructus Immaturus	3 g	*Citrus aurantium* L
Gan Cao	Glycyrrhizae Radix et Rhizoma	5 g	*Glycyrrhiza uralensis* Fisch
Chai Hu	Bupleuri Radix	10 g	*Bupleurum chinense* DC
Dang Gui	Angelicae Sinensis Radix	5 g	*Angelica sinensis* (Oliv.) Diels

### Preparation and quality control of JDHXD

The ratio of Puerariae Radix, Paeoniae Radix Rubra, Persicae Semen, Angelicae Sinensis Radix, Rehmanniae Radix, Carthami Flos, Forsythiae Fructus, Bupleuri Radix, Aurantii Fructus Immaturus and Glycyrrhizae Radix et Rhizoma in JDHXD is 5:10:25:5:15:15:5:10:3:5 (dry weight, units: g). To be specific, after 30 min of immersion within distilled water, all herbs were added in the boiler to boil for a 1-h period. The decoction was later filtered, and herbs were boiled for additional 30 min. Subsequently, the filtrate underwent concentration with the freeze dryer (EYELA FDU-1100, Tokyo, Japan), finally obtaining 1 g dry extract powder from 6.81 g JDHXD liquid. The JDHXD standard dose clinically was 98 g/70 kg. According to the drug compound conversion rate between mice and human beings, drugs were prepared by TCM decoction. Our low dose group was constructed following the clinically relevant dose, under proportional adjustment by the human-to-mouse conversion ratio (9.1). Therefore, there were two dose groups set up, namely, the low-dose JDHXD (12.74 g/kg/d) and high-dose JDHXD (25.48 g/kg/d) groups.

For component analysis of JDHXD, this study employed a Ultra-high Performance Liquid Chromatography (UHPLC)-Orbitrap Fusion High-Resolution Mass Spectrometry system (Palo Alto, USA) using a C18 column (2.1 × 100 mm, 1.7 μm). Mobile phases contained 0.1% formic acid water (A) alongside 100% acetonitrile (B). Our analytical conditions were set according to previous descriptions [32]. Xcalibur™ 4.1 and Compound Discoverer 3.3 were employed for data processing and compound identification separately.

### Animals

The 6–8-week-old male C57BL/6 mice were obtained in Experimental Animal Center of Yanbian University, and raised under the specific pathogen-free (SPF) condition. Our laboratory conditions included: 20–24 °C, 55%−60% humidity, and the 12-h light/dark cycle. Our experimental protocol gained approval from Ethics Committee of Yanbian University (Approval No: YD20250627019; Date: June 27, 2025). Each experimental procedure was performed at Experimental Animal Center of Yanbian University strictly following Guidelines for Welfare and Ethical Review of Laboratory Animals.

### Establishment and grouping of animal models

MI animal models were established through left anterior descending coronary artery (LAD) ligation using the 8–0 silk suture. To be specific, anesthesia was induced in mice using pentobarbital sodium (50 mg/kg). A small-animal ventilator was utilized to maintain respiratory function, skin above the left third intercostal space was incised, and muscles were dissected to expose the heart. Immediate whitening of ventricular wall confirmed successful MI modeling. The perioperative mortality was approximately 10%, mainly caused by acute heart failure or arrhythmia during surgery; no unexpected mortality occurred in the postoperative recovery period.

The sham surgery group (n = 10) underwent identical procedures without LAD ligation. The surviving mouse that received LAD ligation were randomized into model, high-dose JDHXD, low-dose JDHXD, and captopril (20 mg/kg/d) groups, with 10 for every group. Starting 3 days post-MI, animals of JDHXD and captopril groups received daily gavage of JDHXD (at respective concentrations) or captopril for a 28-day period, whereas those of model and sham groups received saline at the same amount.

### Echocardiography

Animals received anesthesia induction using 3% isoflurane, and anesthesia maintenance using 1% isoflurane throughout echocardiography. The high-frequency, high-resolution small-animal ultrasound imaging system (VisualSonics) was adopted for assessing cardiac function. Functional parameters included left ventricular fractional shortening (LVFS), left ventricular ejection fraction (LVEF), left ventricular end-systolic dimension (LVDs), and left ventricular end-diastolic dimension (LVDd) measured based on M-mode images.

### Histopathological analyses

Cardiac tissues underwent 4% paraformaldehyde fixation, gradient ethanol dehydration, paraffin embedding as well as slicing into 4-μm sections to conduct hematoxylin–eosin (HE) alongside Masson's trichrome staining for evaluating fibrosis levels and pathological changes of each group following 28 days. An optical microscope was utilized to examine sections.

### Network pharmacology analysis

To systematically identify therapeutic targets related to MI, three major biomedical databases, TTD, OMIM, and GeneCards, were queried using “MI” as the primary search term. In parallel, potential targets of JDHXD were predicted through SwissTargetPrediction and TCMSP. The overlapping set of genes between JDHXD-related targets and MI-related candidates was then determined with the Venny 2.1 platform, and these shared genes were postulated to mediate the therapeutic efficacy of JDHXD in MI. Furthermore, the DAVID database was utilized for functional category enrichment through Gene Ontology (GO) as well as Kyoto Gene and Genome Encyclopedia (KEGG). This helped to identify closely associated biological processes (BPs), cellular components (CCs), molecular functions (MFs), and important pathways.

### Transcriptome analysis

Trizol Reagent (Invitrogen Life Technologies) was first utilized to separate total RNA. Thereafter, the NanoDrop spectrophotometer (Thermo Scientific) was adopted for measuring RNA content, purity and integrity. Subsequently, total RNA (3 ug) was utilized as the template to prepare RNA samples. Sequencing library generation was accomplished as follows. First, poly-T oligo-attached magnetic beads were used to purify mRNA from total RNA. Then, fragmentation was carried out within the Illumina proprietary fragmentation buffer at increasing temperature with divalent cations. Super Script II and random oligonucleotides were utilized for synthesizing first-strand cDNA, whereas RNase H and DNA Polymerase I were used for preparing second-strand cDNA. Further, the rest overhangs were transformed in blunt ends by the action of exonuclease/polymerase activities, followed by enzyme removal. Ligation of Illumina PE adapter oligonucleotides was accomplished prior to hybridization when the 3′-ends of DNA fragments were adenylated. For selecting cDNA fragments that were 400–500 bp long, the AMPure XP system (Beckman Coulter,Beverly, CA, USA) was adopted for purifying library fragments. Later, the Illumina PCR Primer Cocktail was employed for the selective enrichment of DNA fragments whose bilateral ends were ligated with adaptor molecules through the 15-cycle PCR procedure. The Bioanalyzer 2100 system (Agilent) was subsequently used to purify (AMPure XP system) and quantify products by Agilent high sensitivity DNA assay. Finally, the NovaSeq 6000 platform (Illumina) was utilized to sequence the sequencing library by Shanghai Personal Biotechnology Cp. Ltd.

### Terminal deoxynucleoitidyl transferase (TdT) dUTP Nick-End Labeling (TUNEL)/CTNT assay

Myocardial cell apoptosis was examined by TUNEL apoptosis detection kit (Beyotime). To prepare the TUNEL detection solution, the reaction buffer was supplemented with TdT enzyme at a 1:9 ratio. Cardiac tissue sections were washed once using PBS before co-incubation using TUNEL assay solution and anti-cTNT (1:100). After staining, sections were rinsed thrice using PBS and mounted by the anti-fluorescence quencher medium prior to examination by the fluorescence microscope (Nikon Ti-s).

### Western blotting analysis

Through adopting RIPA buffer that contained protease/phosphatase inhibitors, total protein extracts were obtained in processed myocardial tissues and cardiomyocytes. Protein content determination employed BCA assay (Roche, USA). Denatured samples received separation through 10% SDS-PAGE prior to transfer on PVDF membranes. After 1 h of blockage at 37 °C, these membranes received overnight primary antibody incubation under 4 °C and another 1 h of matched secondary antibody probing at ambient temperature. Immunodetection was implemented with the enhanced chemiluminescence kit (Millipore, USA), while band intensity analysis was completed via Image J software.

### Immunohistochemistry (IHC)

IHC analysis was performed to assess p-PTEN, p-GSK3β(ser9), p-DRP1, and MFN1 expression in myocardial infarction border zones. After deparaffinage and rehydration, cardiac tissue sections were subjected to citrate buffer treatment for antigen retrieval. After 30 min of 3% H₂O₂ treatment for quenching endogenous peroxidase, sections underwent blockage using 5% goat serum, then overnight primary antibody incubation under 4 °C, and later incubation using biotinylated secondary antibodies. After PBS washes, immunoreactivity was visualized via DAB chromogen with hematoxylin counterstaining. The Nikon Eclipse TI-S microscope was adopted for section imaging. Integral optical density (IOD) was analyzed from three random fields for each sample with ImageJ software.

### Immunofluorescence staining

After 4% paraformaldehyde fixation and 30 min of 0.5% Triton X-100 permeabilization at room temperature, cells received 1-h blockage using 3% BSA. Subsequently, cells received overnight primary antibody (anti-cTNT and anti-Vimentin, 1:100) incubation at 4 °C. On next day, samples underwent additional 2 h of secondary antibody incubation (Fluor 594- and 488-conjugated IgG, 1:200), and 15 min of DAPI nuclear staining. The confocal laser scanning microscope (Nikon, Tokyo, Japan) was used for fluorescence imaging.

### Transmission electron microscopy (TEM)

Following 12 h of 2.5% glutaraldehyde fixation, samples were subjected to 2 h of 1% osmium tetroxide post-fixation under 4 °C. After gradient ethanol dehydration, tissues and cells were subjected to epoxy resin embedding for polymerization. The 70-nm ultrathin sections later underwent uranyl acetate and lead citrate staining, before observation through the Hitachi HT7800 TEM (Tokyo, Japan) to assess murine myocardial ultrastructure.

### ROS analysis

ROS contents were measured with the Beyotime ROS Assay Kit. According to respective instructions, TBHP-treated cardiomyocytes were loaded with DCFH-DA (50 μM, 1 h). Cardiac tissue sections were similarly probed using DCFH-DA (100 μM, 1 h). Fluorescent signals were captured using a Nikon Eclipse Ti-S microscope. Three randomly selected fields per sample were analyzed for ROS fluorescence intensity with ImageJ software.

### Flow cytometry of apoptosis

Annexin V-FITC/PI Kit (BD Biosciences, #556,547) was employed for quantifying apoptosis per manufacturer's instructions. After PBS washing, cells were resuspended within the calcium-rich binding buffer (200 μL) before 10 min of Annexin V-FITC staining (5 μL, 25 °C, in dark). Without washing, PI (5 μg/mL) was introduced immediately before analysis. Thereafter, cell apoptosis was distinguished using a BD FACSAria™ III flow cytometer.

### Cell culture and treatment

The 1-day-old Wistar rats were applied in isolating primary neonatal rat cardiomyocytes (NCMs) from their cardiac tissues. Specifically, after rapid excision, hearts were rinsed using d-Hanks solution twice before overnight trypsin digestion at 4 °C. The next day, cardiac tissues underwent digestion using type II collagenase (Sigma-Aldrich), and cell suspension was plated for differential adhesion to separate NCMs from cardiac fibroblasts (CFs) over 2 h. During culture, CF proliferation was suppressed by adding 100 μM 5-bromodeoxyuridine (5-BrdU, Sigma).

NCMs and H9C2 cells received 24 h of JDHXD pretreatment (50, 100, 200 μg/mL). Later, oxidative stress injury was induced on these cells using TBHP. The specific modeling conditions were depicted in previous study [20]. To be specific, NCMs further underwent 100 μM TBHP treatment for 1 h and H9C2 cells underwent 300 μM TBHP treatment for 4 h. Both cells were cultivated within high-glucose DMEM (Gibco) containing 10% FBS (Gibco) as well as 1% penicillin/streptomycin (NCM biotech).

### Cell viability assessment by methyl thiazolyl tetrazolium (MTT) assay

H9C2 and NCM cell lines (1 × 10^4^/well) were inoculated in 96-well plates. After JDHXD (50, 100, 200 μg/mL) and TBHP treatments, respectively, 0.5 mg/mL MTT solution (100 μL) was introduced into every well. Following a 4-h incubation period in dark under 37 °C, MTT solution was eliminated, and DMSO (150 μL) was added. Optical density (OD) was measured at 490 nm.

### MMP test

The JC-1 assay kit (Beyotime Biotechnology, Nanjing, China) was employed for assessing MMP according to specific protocols. At 24 h post-culture, cells underwent 24-h treatment, JC-1 staining, and subsequently examination using a Nikon Ti-s fluorescence microscope.

### Molecular docking

Crystal structures of major components and 3D structures of hub gene proteins were acquired in PubChem and RCSB PDB (http://www.rcsb.org/) databases. AutoDockTools was applied in removing water molecules out of bound ligands, separating bound ligands and adding nonpolar hydrogen molecules. The drug received similar setup in AutoDockTools through water removal and hydrogen addition, and used to be a ligand. If one molecule docked with one protein molecule, it detected its size and later inserted it. Docking parameters and methodologies were prepared. At last, LigPlot + and PyMOL were utilized for visualizing docking results.

### Surface plasmon resonance imaging (SPRi)

We utilized the PlexArray HT A100 SPR equipment for measuring the interaction between the active components in JDHXD and PTEN, according to the previous description [33]. BIA evaluation software (version 3.0) was employed for examining parameters and kinetic curves.

### Adenoviral transfection

Recombinant adenovirus encoding mouse PTEN (rAV-PTEN) and green fluorescent protein-expressing adenovirus (rAV-GFP) obtained in Vigenebio Biosciences (Shandong, China) were utilized for experiments. NCMs were co-incubated for a 12-h duration using viruses at the multiplicity of infection (MOI) of 100 within antibiotic- and serum-free medium. The transfected cells were cultured within fresh medium including 2% FBS for 48 h prior to subsequent treatments.

### Statistical analysis

Quantitative data are presented as mean ± standard deviation (SD). Data were analyzed using GraphPad Prism 8.0 (La Jolla, USA). Between-group differences were assessed via one-way ANOVA, with Bonferroni correction for multiple comparisons (homogeneous variances) and Tamhane’s T2 test (heterogeneous variances). *p* < *0.05* was considered statistically significant.

## Results

### UHPLC/Orbitrap-MS Chromatograms and Network pharmacology for JDHXD

**Fig. 1:**
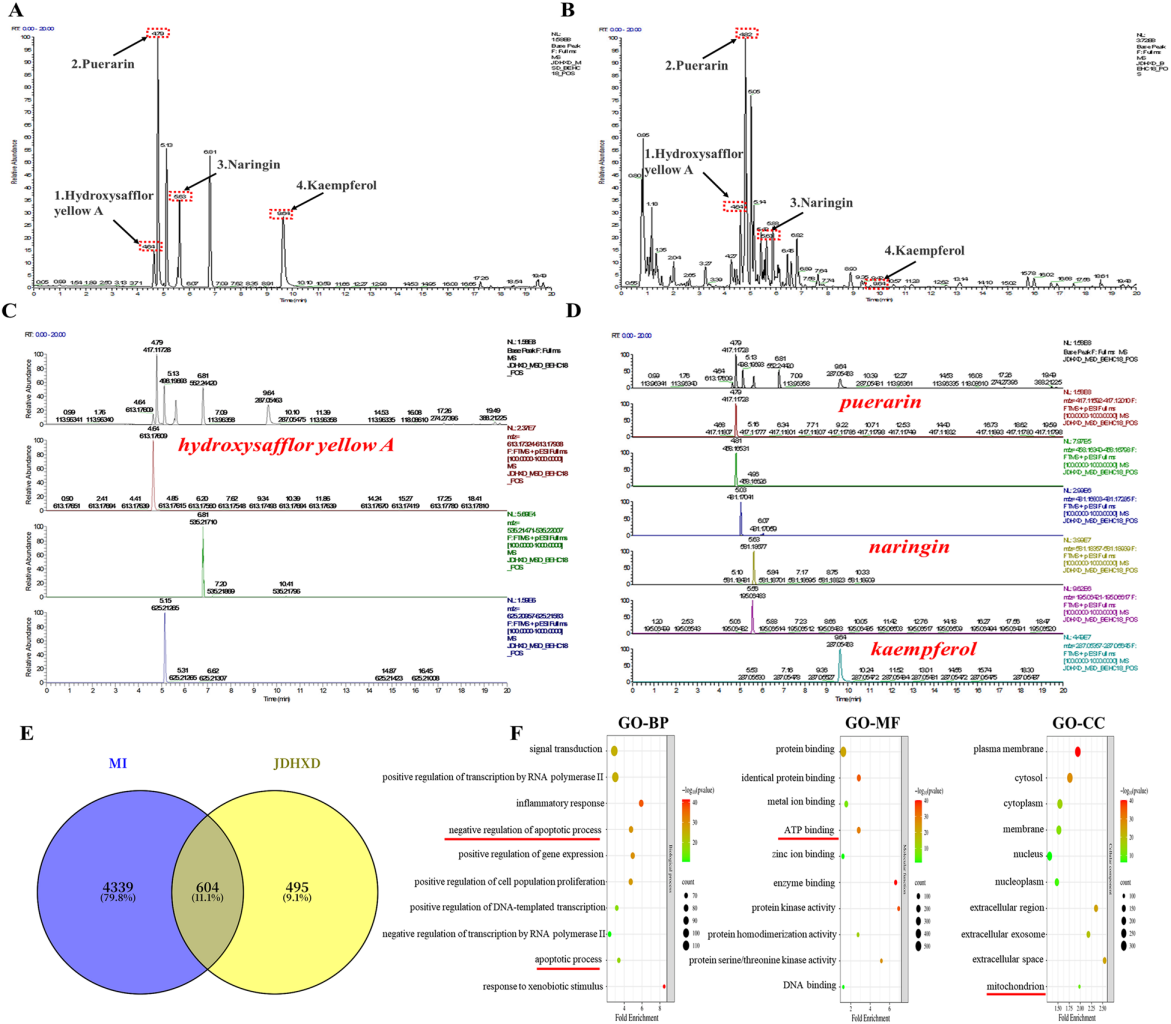
UHPLC/Orbitrap-MS chromatograms and Network pharmacology for JDHXD. **A**–**D** UHPLC/Orbitrap-MS chromatograms for standard substances and JDHXD separately. **E** Venn diagram for overlapping MI-related and JDHXD-related targets. **F** GO enrichment analysis

In this study, we utilized UHPLC-Orbitrap-MS for identifying different substance peaks of JDHXD extracts (Fig. 1A, B). The results in Fig. 1A–D confirmed that the active ingredients of JDHXD used in this study included hydroxysafflor yellow A, puerarin, naringin, and kaempferol. To further clarify the potential pathways of JDHXD in treating MI, a preliminary analysis was conducted using network pharmacology. In total, 4943 potential MI-related genes were obtained from the GeneCards, OMIM, and TTD databases using "MI" as the keyword. Meanwhile, JDHXD-related drug targets were acquired in the TCMSP and SwissTargetPrediction databases. By intersecting MI-related targets with JDHXD-related ones, 604 common targets were obtained (Fig. 1E). Subsequently, GO enrichment (involving BP, CC and MF categories) was conducted on these 604 common targets, as shown in Fig. 1F. The main BP term was associated with negative regulation of apoptosis. The main CC term was mitochondrion. The main MF term primarily comprised ATP binding. As suggested by GO enrichment results, JDHXD may improve myocardial injury post-MI by inhibiting apoptosis through regulating the mitochondrial function.

### JDHXD improves post-MI cardiac function and attenuates cardiomyocyte apoptosis

**Fig. 2:**
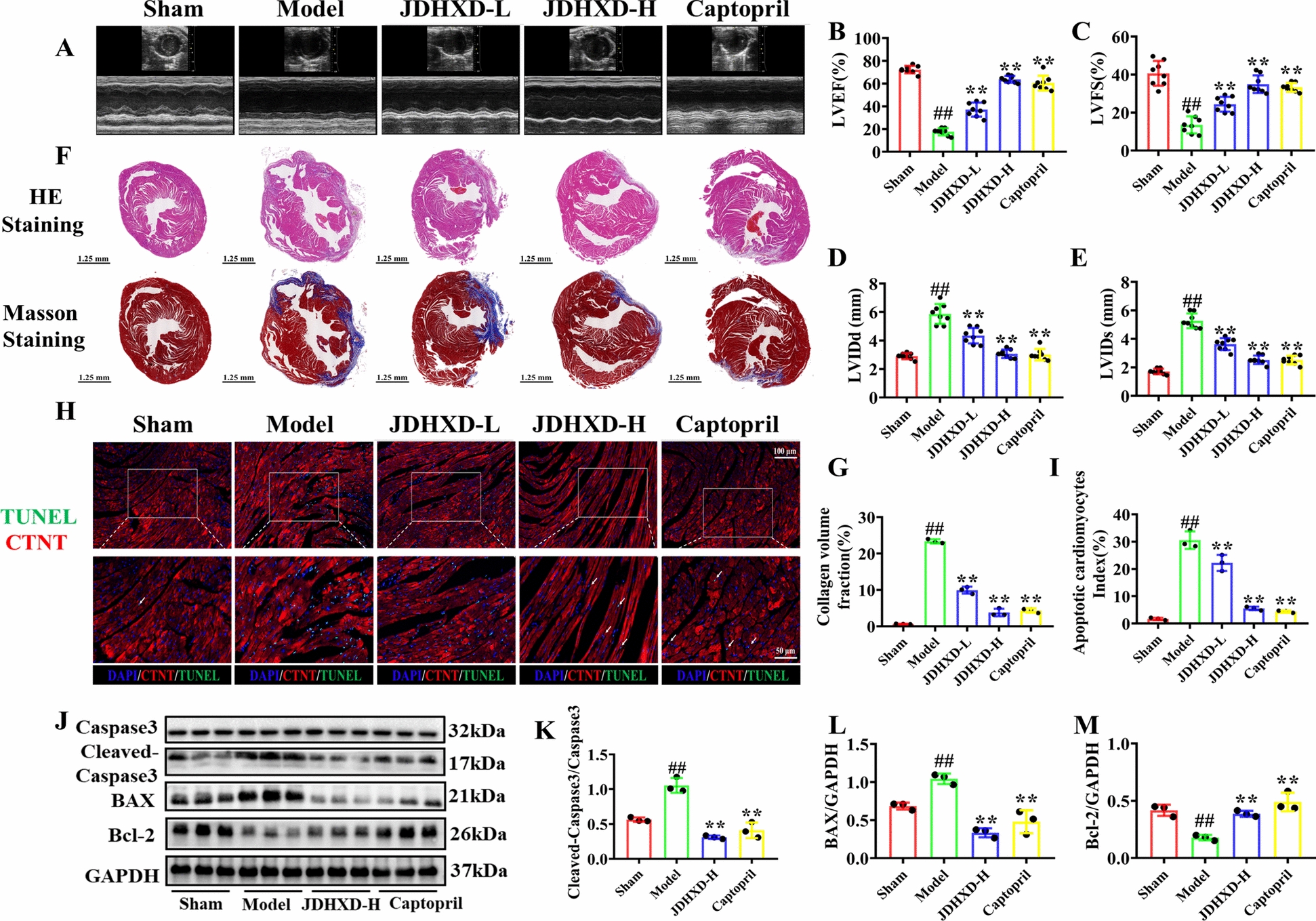
JDHXD enhances post-MI cardiac function and attenuates myocardial apoptosis. **A** Representative M-mode echocardiograms. **B**–**E** LVEF, LVFS, LVDs, and LVDd measurements (n = 8). **F** Representative photomicrographs for HE and Masson staining. **G** Collagen deposition measurement (n = 3). **H**, **I** TUNEL/cTNT staining and apoptotic index (n = 3). Scale bar = 100 μm. **J** Western blotting was performed for analyzing Caspase3, Cleaved-Caspase3, BAX, and BCL-2 protein levels within myocardial tissues. **K**–**M** Quantitative results from **J** (n = 3). Data indicate mean ± SD. ^##^*p* < *0.01* vs. Sham group; **p < *0.01* vs. Model group

To elucidate the cardioprotective effects of JDHXD against MI, the established mouse MI model received daily intragastric administration of JDHXD (12.74 or 25.48 g/kg) or captopril for 28 days postoperatively. Cardiac function and myocardial injury were evaluated using echocardiography and histopathology. Compared with sham controls, MI mice exhibited significantly reduced LVEF and LVFS, alongside increased LVDd and LVDs. Both JDHXD and captopril treatments markedly improved these cardiac parameters (Fig. 2A–E, *p* < *0.01*), and JDHXD enhanced cardiac function dose-dependently. Histological analysis revealed the substantially elevated collagen deposition in MI mice relative to sham controls, whereas JDHXD dose-dependently attenuated fibrosis (Fig. 2F, G, *p* < *0.01*). To further verify JDHXD’s protective effect on cardiomyocytes after MI, TUNEL/cTnT double staining was performed to assess cardiomyocyte apoptosis. As shown, cardiomyocyte apoptosis was markedly increased following MI. JDHXD reduced cardiomyocyte apoptosis in a dose‑dependent manner, and high‑dose JDHXD exhibited a prominent anti‑apoptotic effect comparable to captopril (Fig. 2H, I, *p* < *0.01*). Western blotting further confirmed that MI upregulated Cleaved‑Caspase3 and BAX, while downregulating Bcl‑2 (Fig. 2J–M, *p* < *0.01*). Consistently, high‑dose JDHXD significantly reversed these apoptotic markers, achieving a therapeutic effect equivalent to captopril. Collectively, JDHXD alleviates post-MI cardiac dysfunction primarily through attenuating cardiomyocyte apoptosis.

### Network pharmacology and transcriptomic analysis for potential targets of JDHXD in MI

**Fig. 3:**
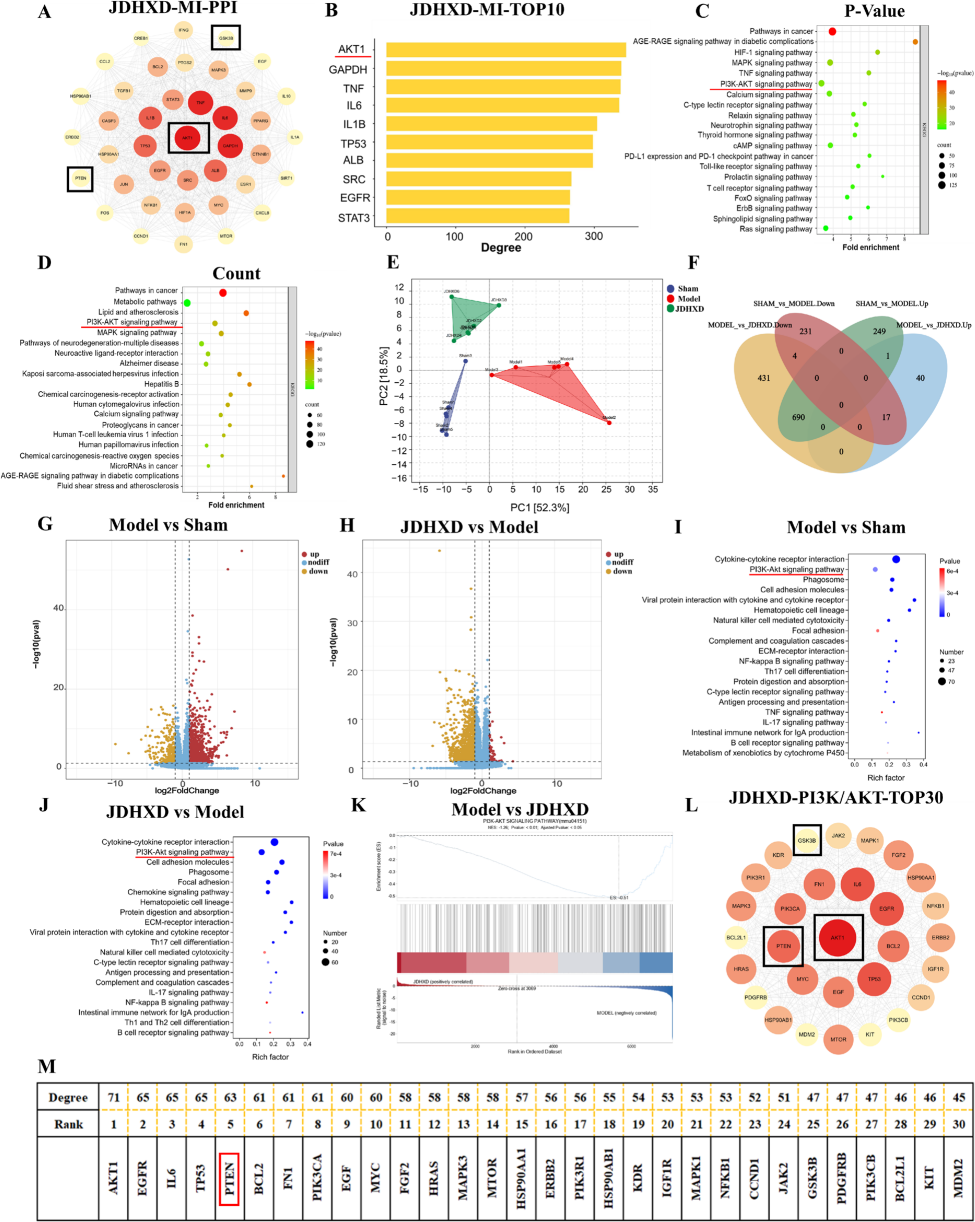
Network pharmacology and transcriptomic analysis of potential targets of JDHXD in MI. **A** PPI network of common targets between JDHXD and MI. **B** The top 10 core common targets of JDHXD and MI. **C**, **D** KEGG enrichment analysis of common targets based on p-value (**C**) and count (**D**). **E** Principal component analysis (PCA) of transcriptomes from Sham, Model, and JDHXD groups. **F** Venn diagram of differentially expressed genes among three groups. **G**, **H** Volcano plots of DEGs in Model vs Sham (**G**) and JDHXD vs Model (**H**) groups. **I**, **J** KEGG enrichment analysis of differentially expressed genes in Model vs Sham (**I**) and JDHXD vs Model (**J**) groups. **K** GSEA enrichment of the PI3K/AKT signaling pathway (mmu04151). **L**, **M** The top 30 common targets involved in the JDHXD-regulated PI3K/AKT pathway

By utilizing Cytoscape software, topology analysis was conducted on JDHXD and MI core metrics, and the 604 common targets were input in STRING database to draw the PPI network. From Fig. 3A, B, PPI plots for the degree values of the top 40 core targets of JDHXD and MI were established, and the top 10 were ranked, which were AKT1, GAPDH, TNF, IL-6, IL1B, TP53, ALB, SRC, EGFR and STAT3, respectively. KEGG enrichment was further conducted to explore the pathways of JDHXD in MI. And the top 20 significant pathways were screened according to the *P-value* and the Count of targets, suggesting that PI3K/AKT pathway was closely related to JDHXD (Fig. 3C, D).

To verify the main approaches by which JDHXD improved MI, we carried out transcriptome analysis on heart tissue samples (n = 6). Principal component analysis (PCA, Fig. 3E) results illustrated significant differences across three groups, suggesting that drug intervention and modeling influence transcription patterns. In addition, compared with the Sham group, the Model group displayed a more pronounced upward shift in gene expression, while that in the JDHXD group returned to their normal levels, demonstrating that JDHXD facilitates normal gene levels in MI hearts. There were altogether 707 differential genes obtained across three groups, and JDHXD reversed the up-regulation of 17 genes whereas the down-regulation of 690 genes (Fig. 3F). Relative to the Sham group, there were 940 and 252 genes showing up-regulation and down-regulation, respectively, in the Model group (Fig. 3G); relative to the Model group, there were 58 up-regulated and 1125 down-regulated genes in the JDHXD group (Fig. 3H). The differential genes in the Sham vs. Model and Model vs. JDHXD groups were subjected to KEGG enrichment analyses, respectively. As shown in Figure Fig. 3I, J, the differential genes among the Sham, Model and JDHXD groups were all closely related to the PI3K/AKT pathway (mmu04151). Further GSEA results indicated that the JDHXD group significantly activated the PI3K/AKT pathway relative to the Model group (Fig. 3K).

All these results suggest that JDHXD may improve myocardial injury post-MI through modulating the PI3K/AKT pathway. Thereafter, degree values of JDHXD and the targets related to this pathway were further ranked, indicating that JDHXD may significantly regulate PTEN and GSK3β to exert its effects (Fig. 3L, M).

### JDHXD improves oxidative stress-induced mitochondrial fission/fusion disorder post-MI through suppressing PTEN/AKT/GSK3β pathway

**Fig. 4:**
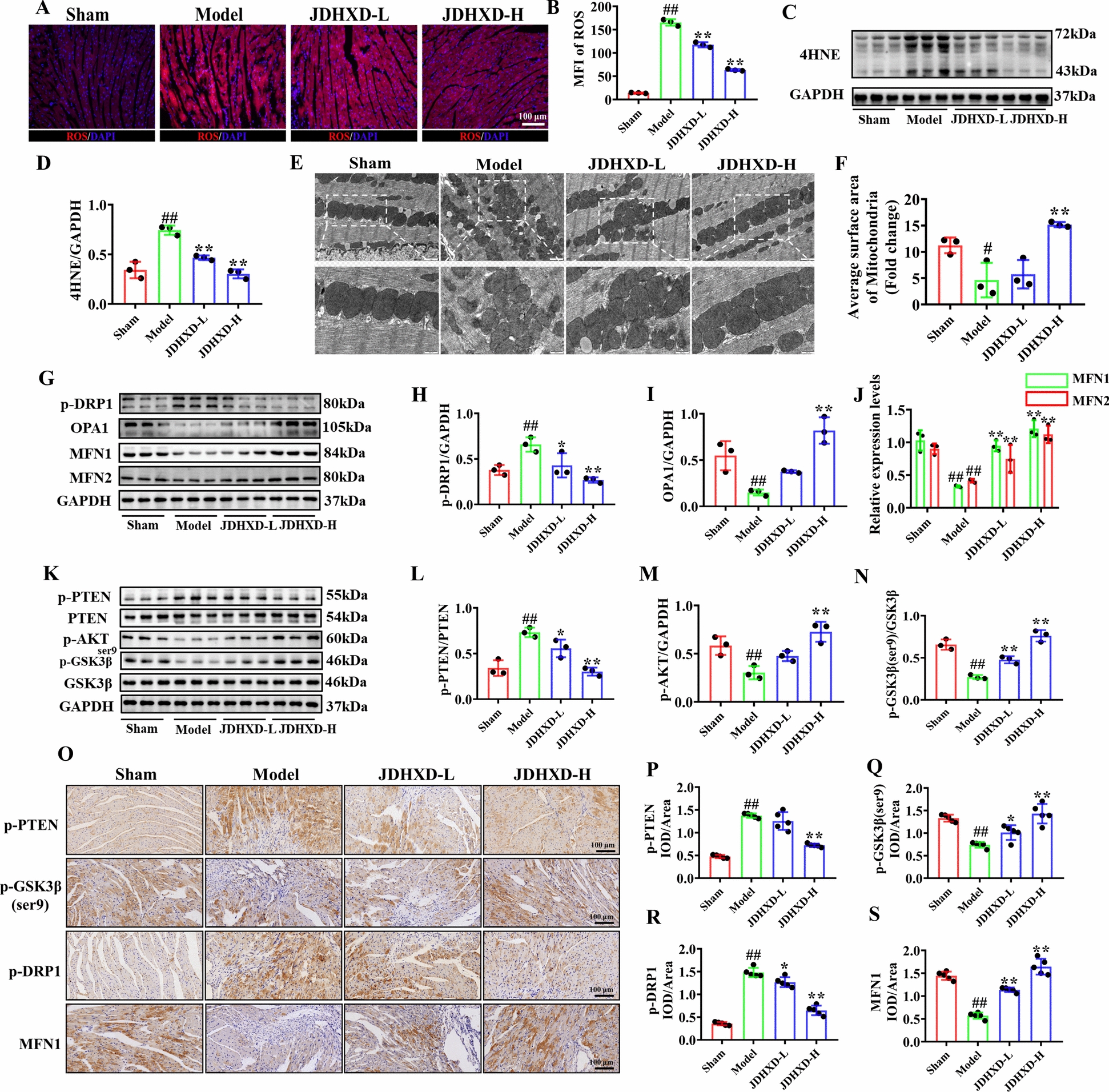
JDHXD improves oxidative stress-induced mitochondrial fission/fusion disorder after MI through suppressing the PTEN/AKT/GSK3β pathway. **A**, **B** Typical images of ROS staining and quantitative ROS levels inside myocardial tissues (n = 3). Scale bar = 100 μm. **C**, **D** 4-HNE level within cardiac tissues was examined through Western blotting (n = 3). **E**, **F** Mitochondrial morphology and surface area in cardiac tissues were examined by TEM (n = 3). **G**–**J** Mitochondrial fission/fusion regulatory protein levels (p-DRP1, OPA1, MFN1, MFN2) inside cardiac tissues (n = 3). **K**–**N** Expression levels of PTEN/AKT/GSK3β pathway-related proteins in cardiac tissues (n = 3). **O**–**S** p-PTEN, p-GSK3β(ser9), p-DRP1, and MFN1 protein levels inside cardiac tissues were measured by IHC (n = 5). Scale bar = 100 μm. Data indicate mean ± SD. ^##^*p* < *0.01* vs. Sham group; *p < *0.05,* **p < *0.01* vs. Model group

To investigate whether JDHXD inhibited post-MI oxidative stress, ROS contents in myocardial tissues were quantified through ROS staining. Our results suggested markedly increased ROS contents of MI model versus sham groups, while JDHXD dose-dependently reduced ROS levels (Fig. 4A, B, *p* < *0.01*). Western blotting analysis revealed that 4-HNE, a lipid peroxidation biomarker, was markedly up-regulated in model group, whereas JDHXD dose-dependently inhibited its expression (Fig. 4C, D, *p* < *0.01*). Oxidative stress injury post-MI results in mitochondrial fission/fusion disorder, abnormal mitochondrial fission and fragmentation, and mitochondrial fusion disorder, which then reduces the mitochondrial surface area and causes rupture of intimal crists, thereby promoting secretion of Cytc as well as additional pro-apoptotic factors in cytoplasm and inducing cardiomyocyte apoptosis. Using TEM, we observed the effects of JDHXD on mitochondria post-MI and found that the morphology of the mitochondria was abnormal (reduced body surface area and crista fragmentation), and JDHXD improved the morphology of mitochondria dose-dependently after oxidative stress induction, significantly increasing their surface area (Fig. 4E, F, *p* < *0.01*). To determine whether JDHXD improved oxidative stress-induced mitochondrial fission/fusion disorder, Western blotting assay was carried out for analyzing protein levels. The results indicated the significantly elevated p-DRP1 whereas reduced OPA1, MFN1 and MFN2 levels post-MI. JDHXD dose-dependently reversed the abnormal changes of these protein levels (Fig. 4G–J, *p*  < *0.01*). To further clarify the specific mechanism by which JDHXD protected against myocardial injury, Western blotting was further performed for detecting levels of the PTEN/AKT/GSK3β pathway proteins. From Fig. 4K–N, p-PTEN level significantly elevated during MI. JDHXD could inhibit its level dose-dependently and promote downstream AKT phosphorylation, resulting in the inactivation of GSK3β (*p* < *0.01*). The IHC results were consistent (Fig. 4O–S, *p* < *0.01*). Based on the above results, JDHXD mitigates oxidative stress-mediated mitochondrial injury during MI partially through suppressing the PTEN/AKT/GSK3β pathway.

### JDHXD attenuates TBHP-induced cardiomyocyte apoptosis

**Fig. 5:**
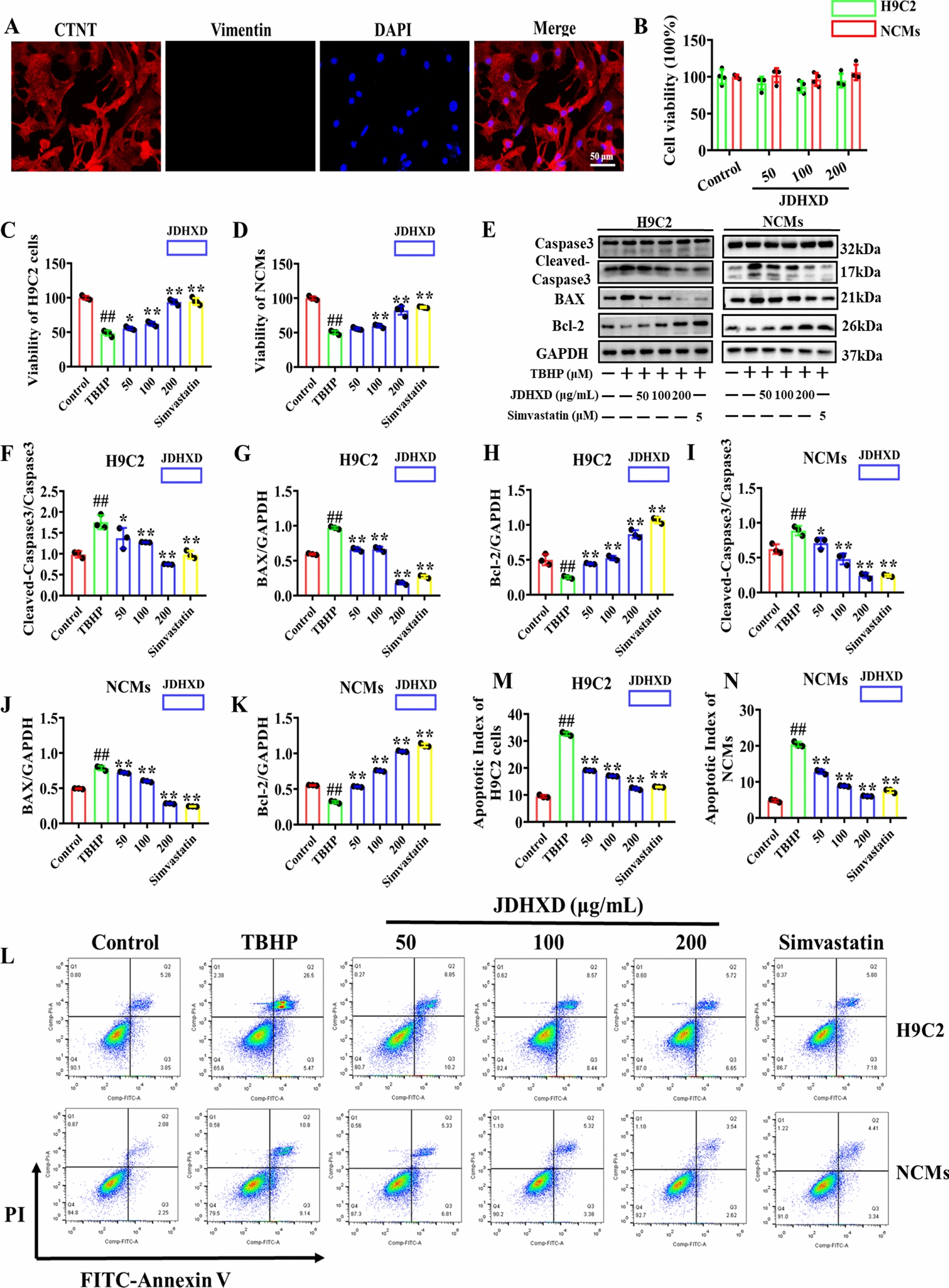
JDHXD attenuates TBHP-induced apoptosis in cardiomyocytes. **A** NCMs identification was completed through immunofluorescence analysis. Scale bar = 50 μm. **B** Cytotoxicity assessment of JDHXD at gradient concentrations in H9C2 cells and NCMs after 24-h exposure was performed by MTT assay (n = 4). **C**, **D** H9C2 and NCM cell viability following JDHXD exposure at gradient concentrations (n = 4). **E** The apoptosis-associated protein levels in H9C2 cells and NCMs were determined through Western blotting. **F**–**K** Quantitative analysis of **E** (n = 3). **L**–**N** Flow cytometry and Annexin V-FITC/PI double-staining were conducted for quantifying H9C2 cell and NCM apoptosis rates (n = 3). Data indicate mean ± SD. ^##^*p* < *0.01* vs. control group; *p < *0.05*, **p < *0.01* vs. TBHP-treated group

To evaluate JDHXD’s role in resisting cardiomyocyte apoptosis in vitro, TBHP was used for simulating post-MI oxidative stress-induced apoptosis. Primary NCMs were isolated from neonatal Wistar rat pups for further investigation. cTNT marker-based immunofluorescence staining was first employed to identify NCMs, while vimentin marker for CFs was applied to exclude CFs. As shown in Fig. 5A, the isolated cells exhibited cTNT-positive but vimentin-negative staining, confirming that they were NCMs. We then determined whether JDHXD induced cytotoxicity to cardiomyocytes. The MTT assay demonstrated that JDHXD induced no cytotoxicity within the tested concentration range (Fig. 5B). As shown in Fig. 5C, D, TBHP significantly reduced cardiomyocyte viability compared with control group, while JDHXD at 50, 100, and 200 μg/mL improved cell survival dose-dependently (*p* < *0.01*). Analysis of apoptosis-related proteins (Fig. 5E–K, *p* < *0.01*) revealed that TBHP markedly up-regulated cleaved caspase-3 and BAX and down-regulated Bcl-2, and such effects could be dose-dependently abolished by JDHXD. Flow cytometry data (Fig. 5L–N, *p* < *0.01*) demonstrated the increased proportions of H9C2 cells and NCMs in Q2 (late apoptosis) and Q3 (early apoptosis) quadrants after TBHP exposure compared with the control group. JDHXD (50–200 μg/mL) dose-dependently attenuated these changes, and similar protection was observed in the simvastatin positive control group. Collectively, these findings indicate that JDHXD suppresses TBHP-induced cardiomyocyte apoptosis.

### JDHXD prevents against TBHP-induced mitochondrial injury of cardiomyocytes through suppressing PTEN/AKT/GSK3β pathway

**Fig. 6:**
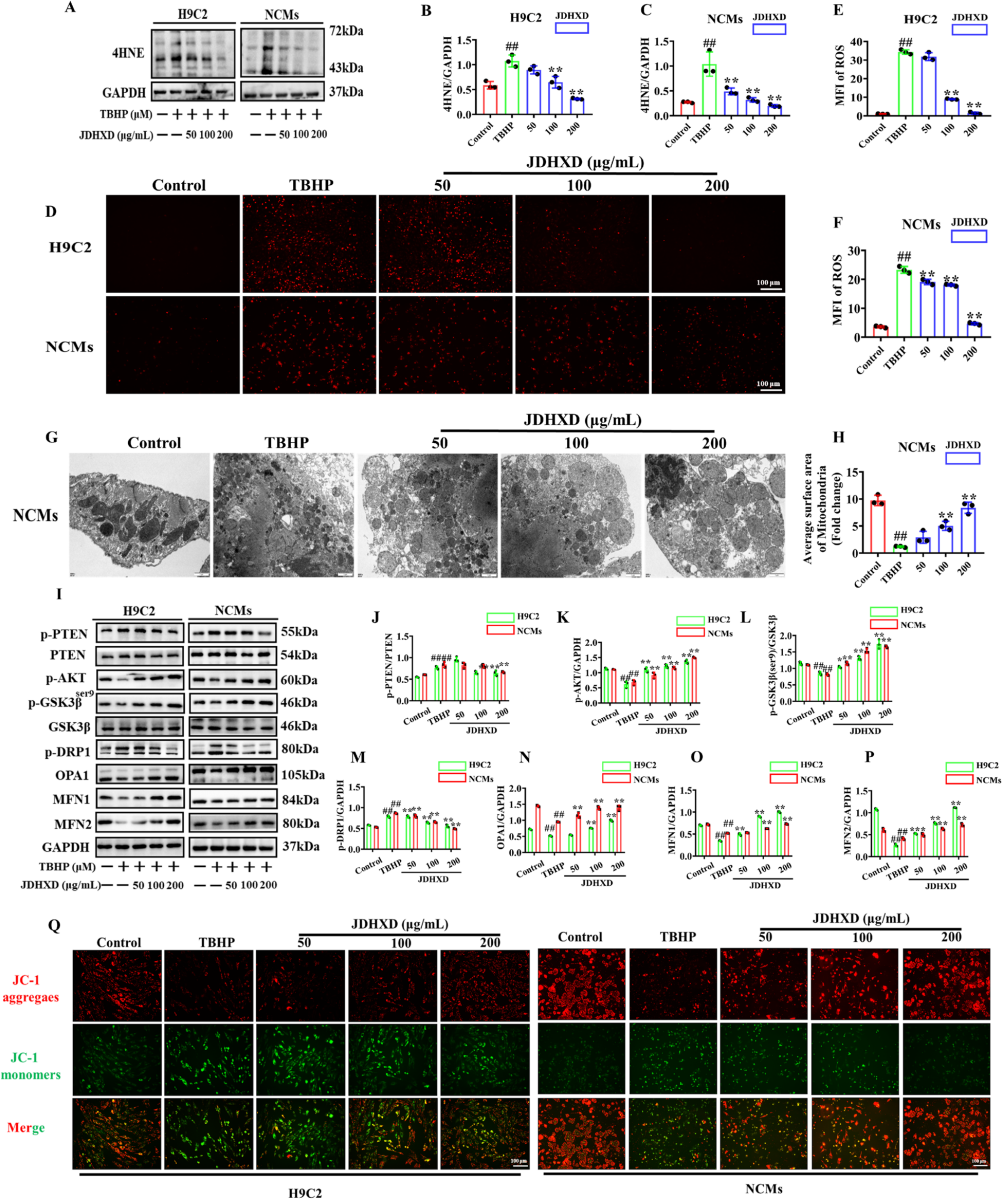
JDHXD protects against TBHP-induced mitochondrial injury by mitigating oxidative stress in cardiomyocytes, concurrently inhibiting PTEN pathway activation. **A** The 4-HNE expression inside H9C2 cells and NCMs were examined through Western blotting analysis. **B**, **C** Quantification of **A** (n = 3). **D**–**F** ROS contents inside H9C2 cells and NCMs (n = 3). Scale bars = 100 μm. **G**, **H** Mitochondrial morphology and surface area in cardiac tissues were measured by TEM (n = 3). **I** Expression levels of PTEN/AKT/GSK3β pathway-related proteins and mitochondrial fission/fusion regulatory proteins (p-DRP1, OPA1, MFN1, MFN2) in cardiomyocytes were determined through Western blotting analysis. **J**–**P** Quantitative analysis of **I** (n = 3). **Q** MMP damage was determined using JC-1 probe within H9C2 cells and NCMs. Data indicate mean ± SD. ^##^*p* < *0.01* vs. control group; *p < *0.05*, **p < *0.01* vs. TBHP-treated group

To elucidate JDHXD’s protective mechanisms in TBHP-induced mitochondrial injury, Western blotting analysis revealed significantly elevated 4-HNE levels in TBHP-treated versus control groups. JDHXD treatment markedly reduced 4-HNE levels in both H9C2 and NCMs (Fig. 6A–C, *p* < *0.01*). ROS assays demonstrated that TBHP increased intracellular ROS levels, while JDHXD dose-dependently suppressed ROS accumulation (Fig. 6D–F, *p* < *0.01*). TEM showed that after oxidative stress injury, the abnormal mitochondrial fission in cardiomyocytes significantly increased, and mitochondria became smaller and rounder. JDHXD improved the abnormal fission/fusion of damaged mitochondria dose-dependently, which specifically manifested as an increase in the surface area of mitochondria (Fig. 6G, H, *p* < *0.01*). As confirmed by subsequent Western blotting analysis, JDHXD modulated levels of PTEN/AKT/GSK3β signaling pathway proteins and mitochondrial fission/fusion regulators (p-DRP1, OPA1, MFN1, MFN2) (Fig. 6I–P, *p* < *0.01*). Abnormal mitochondrial fission/fusion caused by ROS accumulation can change mitochondrial membrane permeability, thereby reducing MMP. In this study, we conducted JC-1 staining for clarifying JDHXD’s mitochondrial protection after oxidative stress injury. As shown in Fig. 6Q, oxidative stress damage led to changes in mitochondrial permeability and disorders in MMP (reduced red fluorescence whereas enhanced green fluorescence intensities). JDHXD dose-dependently reversed this trend (elevated red fluorescence but declined green fluorescence intensities). Taken together, JDHXD prevents TBHP-induced mitochondrial injury of cardiomyocytes partially by inhibiting the PTEN/AKT/GSK3β signaling pathway.

### Suppression on the PTEN does not potentiate the protection of JDHXD against TBHP-induced mitochondrial injury of cardiomyocytes

**Fig. 7:**
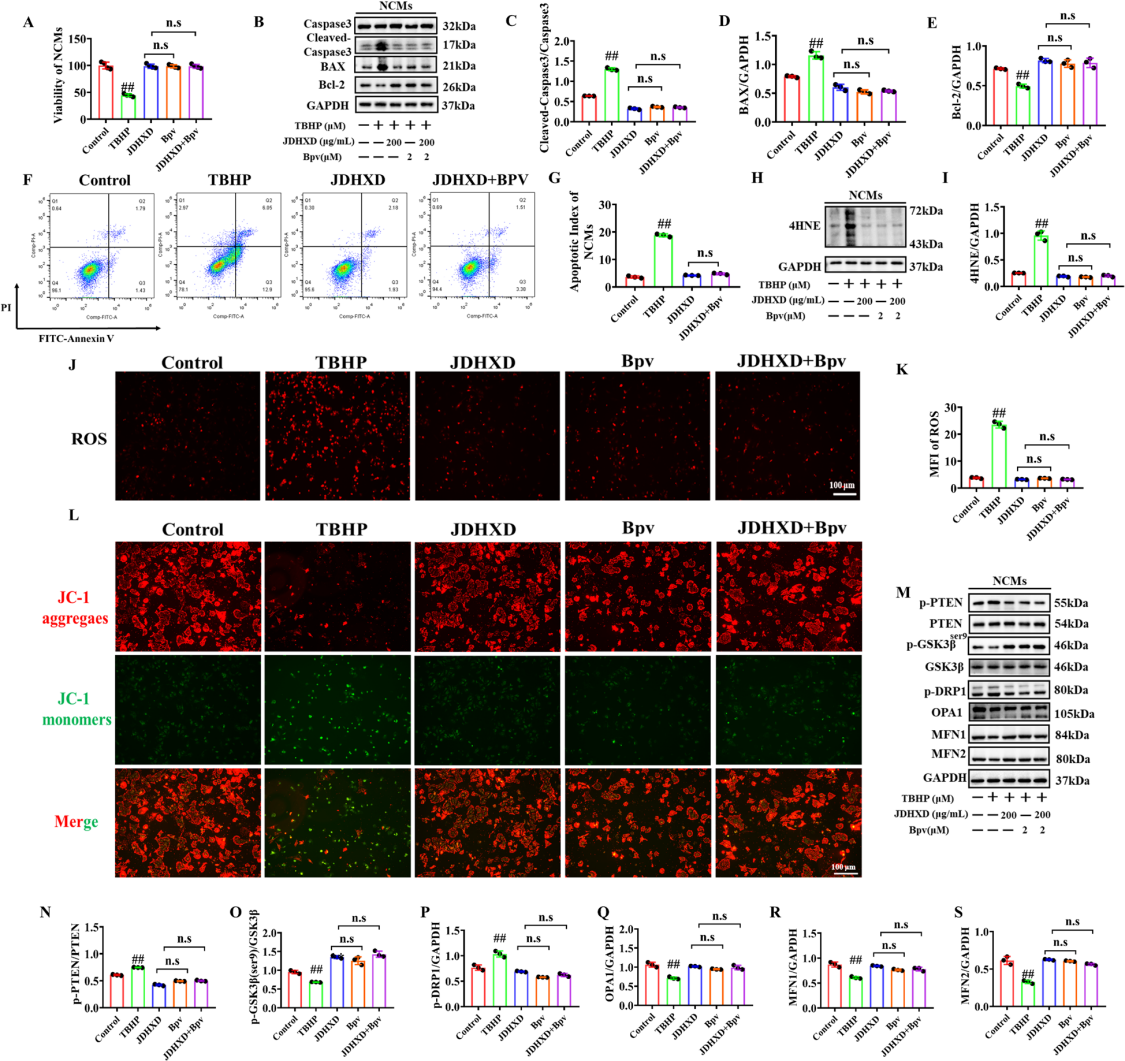
Suppression of the PTEN does not potentiate the protection of JDHXD from TBHP-induced mitochondrial injury of cardiomyocytes. **A** Cell viability of NCMs (n = 4). **B** Western blotting assay was performed for detecting apoptosis-related protein levels. **C**–**E** Quantification of **B** (n = 3). **F**, **G** Flow cytometry and Annexin V-FITC/PI double-staining were conducted to quantify NCM apoptosis rate (n = 3). **H**, **I** 4-HNE level within NCMs was analyzed through Western blotting (n = 3). **J**, **K** ROS contents within NCMs (n = 3). Scale bar = 100 μ m. **L** JC-1 probe was used in analyzing MMP damage of NCMs. **M** Expression levels of PTEN/GSK3β pathway-related proteins and mitochondrial fission/fusion regulatory proteins (p-DRP1, OPA1, MFN1, MFN2) in cardiomyocytes were determined through Western blotting analysis. **N**–**S** Quantitative analysis of **M** (n = 3). Data indicate mean ± SD. ^##^*p* < *0.01* vs. control group. n.s: non-significance

To analyze how PTEN signaling inhibition affected JDHXD's protection from TBHP-mediated NCM apoptosis, 2 μM Bpv was administered. Cell viability assays (Fig. 7A) showed the evidently decreased viability in TBHP versus control groups, while JDHXD monotherapy and JDHXD + Bpv combination restored cell viability without statistical difference between these two groups. Western blotting for analyzing apoptosis-associated proteins (Fig. 7B) and quantifying their levels (Fig. 7C–E) revealed that TBHP exposure increased cleaved caspase-3 and BAX contents whereas down-regulated Bcl-2. Both treatments comparably abolished these alterations without significant intergroup differences. Flow cytometry and Annexin V-FITC/PI double-staining (Fig. 7F, G) confirmed TBHP-induced apoptosis, which was equivalently attenuated by JDHXD alone or combined with Bpv.

To further clarify PTEN’s effect on JDHXD in improving oxidative stress-induced mitochondrial injury, we assessed 4-HNE level within NCMs via Western blotting assay (Fig. 7H, I). The results demonstrated that TBHP significantly increased 4-HNE expression relative to control group. Besides, JDHXD and JDHXD + Bpv treatments reversed this alteration, with no significant difference. Consistent results could be obtained from ROS level detection (Fig. 7J, K). Subsequently, JC-1 probe analysis revealed MMP impairment (Fig. 7L). The TBHP-treated group exhibited a decreased red-to-green fluorescence ratio (decreased red fluorescence and increased green fluorescence intensities), indicating MMP disruption. Both JDHXD and JDHXD + Bpv treatments reversed this trend (increased red fluorescence and decreased green fluorescence intensities), suggesting MMP preservation, though without significant intergroup difference. Further Western blotting analysis of PTEN/GSK3β pathway proteins and mitochondrial fission/fusion regulators (p-DRP1, OPA1, MFN1, MFN2) (Fig. 7M–S) showed that activation of TBHP-induced PTEN pathway (reduced p-GSK3β(ser9) expression) and mitochondrial dynamic imbalance (elevated p-DRP1; reduced OPA1, MFN1, MFN2) were similarly rescued by JDHXD and JDHXD + Bpv, with no statistical intergroup difference. Collectively, these data indicate that suppression of the PTEN does not potentiate the protection of JDHXD against TBHP-induced mitochondrial injury in cardiomyocytes.

### PTEN overexpression partially attenuates JDHXD’s protection from TBHP-induced mitochondrial injury of cardiomyocytes

**Fig. 8:**
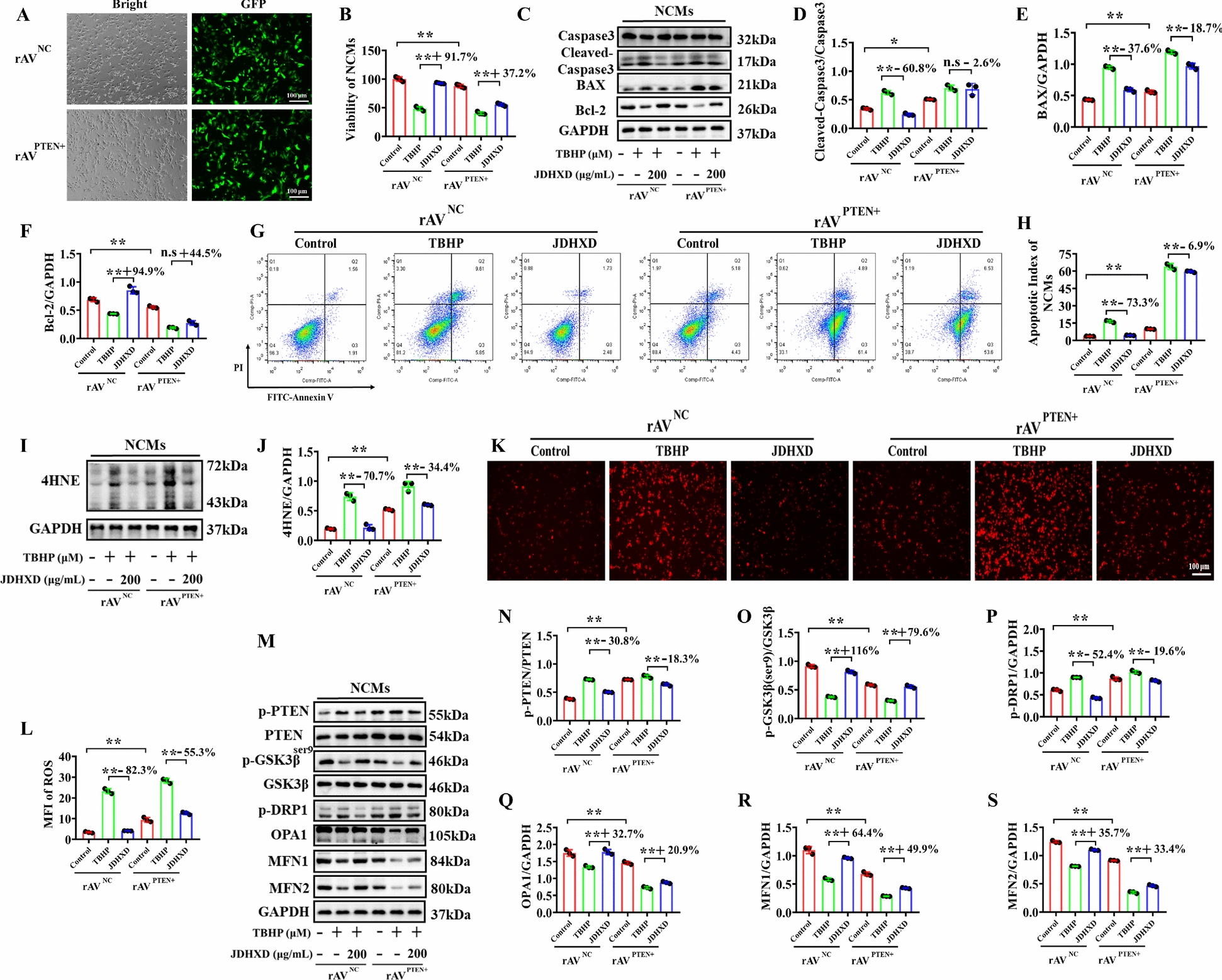
PTEN overexpression partially attenuates JDHXD’s protection from TBHP-induced mitochondrial injury of cardiomyocytes. **A** Bright-field images and GFP fluorescence of NCMs transfected with rAV-GFP or rAV-PTEN. Scale bar = 200 μm. **B** Cell viability of NCMs (n = 4). **C** Apoptosis-associated protein contents, like Cleaved-Caspase3, BAX, and Bcl-2 in NCMs, were measured through Western blotting. **D**–**F** Quantitative analysis of **C** (n = 3). **G**, **H** Flow cytometry and Annexin V-FITC/PI double-staining were conducted for quantifying NCM apoptosis rate (n = 3). **I**, **J** 4-HNE level inside NCMs was analyzed through Western blotting (n = 3). **K**–**L** ROS contents within NCMs (n = 3). Scale bar = 100 μm. **M** Levels of PTEN/GSK3β pathway-related proteins and mitochondrial fission/fusion regulatory proteins (p-DRP1, OPA1, MFN1, MFN2) in cardiomyocytes were examined through Western blotting. **N**–**S** Quantitative analysis of **M** (n = 3). Data indicate mean ± SD. *p < *0.05*, **p < *0.01*. n.s: non-significance

To clarify the PTEN dependence of JDHXD-conferred cardioprotection, mouse PTEN was overexpressed in NCMs using a rAV-PTEN. Robust GFP fluorescence was observed in rAV-PTEN-transfected NCMs (Fig. 8A). Moreover, the Western blotting results showed that PTEN was significantly up-regulated in the cells transfected with rAV-PTEN (Fig. 8M, N, *p* < *0.01*). Collectively, these findings indicate the successful construction of the PTEN-overexpression cardiomyocytes. Key findings revealed that compared with rAV-GFP controls, PTEN overexpression significantly reduced cell viability in sham-operated NCMs (Fig. 8B), and partially abrogated the anti-apoptotic protection of JDHXD against TBHP stimulation, which manifested as the weakened suppression on pro-apoptotic proteins (cleaved caspase-3, BAX) and impaired up-regulation of anti-apoptotic Bcl-2 (Fig. 8C–F). Quantitative Annexin V-FITC/PI analysis consistently demonstrated that PTEN overexpression substantially diminished the JDHXD-mediated apoptosis reduction (Fig. 8G, H). This study next interrogated whether JDHXD mitigated oxidative stress-induced mitochondrial injury through suppressing PTEN/GSK3β pathway. As anticipated, PTEN-overexpressing (PTEN-OE) sham-operated cardiomyocytes exhibited the heightened oxidative stress susceptibility versus controls. Critically, according to Fig. 8I–L, PTEN-OE sham cells showed significantly elevated 4-HNE and ROS levels, while in TBHP-challenged NCMs, PTEN overexpression partially attenuated JDHXD's suppression on oxidative stress. Western blotting analysis of the GSK3β pathway and mitochondrial fission/fusion regulators (p-DRP1, OPA1, MFN1, MFN2) (Fig. 8M, O–S) further revealed that PTEN-OE sham cells displayed reduced p-GSK3β(Ser9) expression alongside increased p-DRP1 level and decreased OPA1, MFN1 and MFN2 levels. In TBHP-insulted NCMs, PTEN overexpression partially attenuated the effects of JDHXD on suppressing GSK3β pathway and normalizing mitochondrial dynamics-associated proteins. From the above results, JDHXD exerts its protective effects against TBHP-mediated mitochondrial injury of cardiomyocytes partially via inhibiting PTEN.

### Predicting active compounds of JDHXD in the PTEN/AKT/GSK3β signaling pathway

**Fig. 9:**
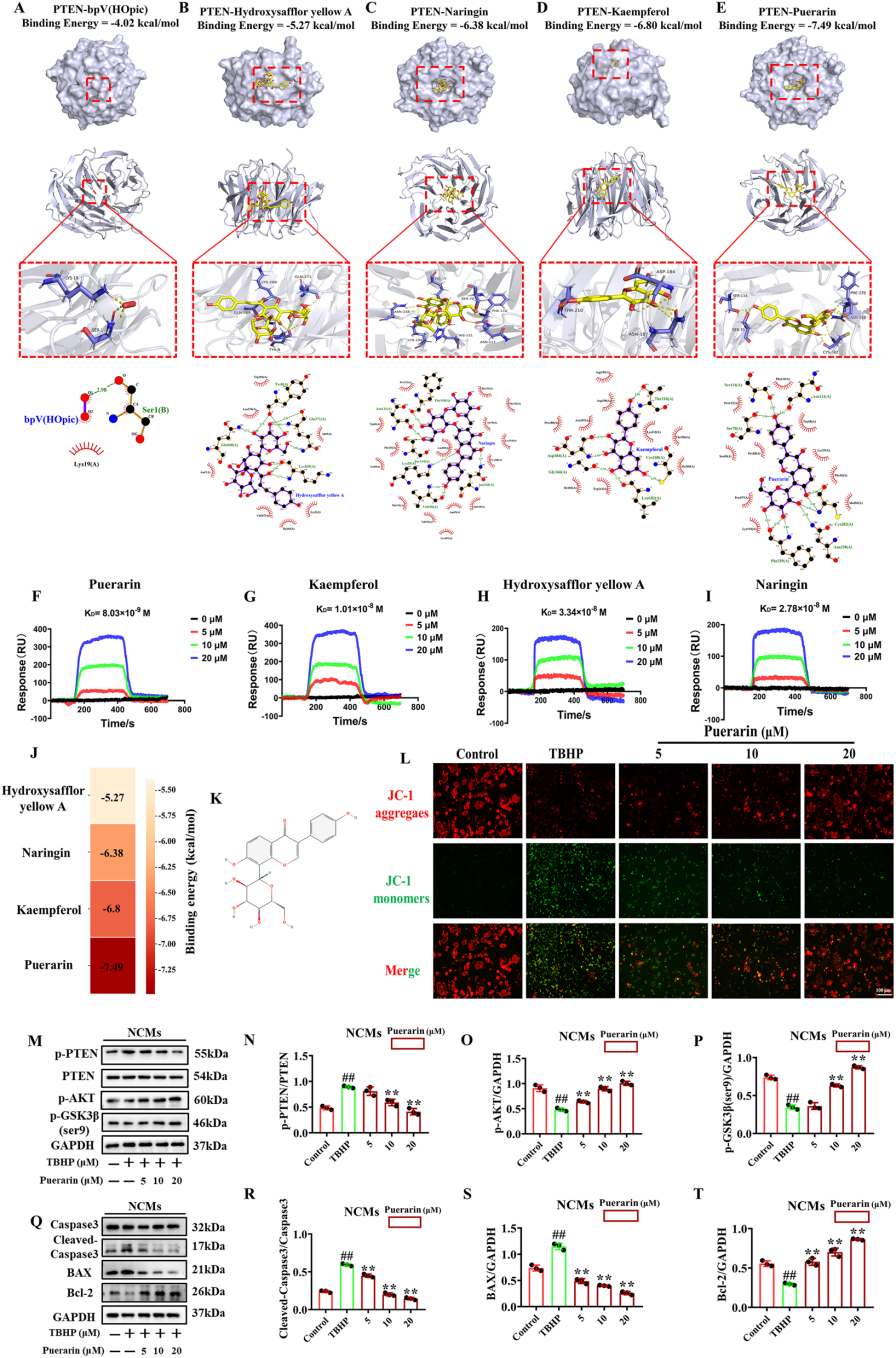
Predicting active compounds of JDHXD in the PTEN/AKT/GSK3β signaling pathway. **A** Bpv(HOpic)-PTEN. **B** Hrdroxysafflor yellow A-PTEN. **C** Naringin-PTEN. **D** Kaempferol-PTEN. **E** Puerarin-PTEN. **F**–**I** SPR fitting curves showing puerarin, kaempferol, hrdroxysafflor yellow A and naringin at varying concentrations to PTEN. **J** Molecular docking and binding energy sequencing. **K** Molecular formula of Puerarin. **L** JC-1 probe was used in analyzing MMP damage of NCMs. **M** Expression levels of PTEN/AKT/GSK3β pathway-related proteins in cardiomyocytes were determined through Western blotting analysis. **N**–**P** Quantitative analysis of **M** (n = 3). **Q** Apoptosis-associated protein contents, like Caspase3, Cleaved-Caspase3, BAX, and Bcl-2 in NCMs, were measured by Western blotting analysis. **R**–**T** Quantitative analysis of **Q** (n = 3). Data indicate mean ± SD. ^##^*p* < *0.01* vs. control group. *p < *0.05*, **p < *0.01* vs. TBHP-treated group

According to UHPLC/Orbitrap-MS of JDHXD, this study conducted molecular docking for exploring the effective components in JDHXD that suppressed the PTEN protein for resisting myocardial injury post-MI. Molecular docking is capable of predicting possible efficacy of drug components according to the respective hub gene-binding potential. In this work, top-ranked compounds, kaempferol, naringin, hrdroxysafflor yellow A and puerarin, were screened and docked with PTEN protein (PDB ID: 8 × 3 s). PyMOL and LigPlus were adopted for visualizing the binding mode to investigate core target-containing compounds for their structural basis. Figure 9A–E show the binding energy, three-dimensional binding and hydrogen bond plots of Bpv, hydroxyl safflower A, puerarin, naringin and kaempferol with PTEN protein. From our findings, the binding energies of hydroxysafflower A, puerarin, narnarin, and kaempferol to PTEN were − 5.27, − 7.49, − 6.38, and − 6.8 kcal·mol^−1^, separately, markedly larger than that of the PTEN inhibitor Bpv (− 4.02 kcal·mol^−1^). We also conducted SPR analysis for examining the protein-compound interactions. As a result, kaempferol, naringin, hrdroxysafflor yellow A and puerarin could directly target PTEN dose-dependently (Fig. 9F–I). And the surface plasmon resonance (SPR) showed the KD values of hydroxysafflor yellow A, puerarin, naringin, and kaempferol with PTEN, respectively, were 3.34 × 10^–8^, 8.03 × 10^–9^, 2.78 × 10^–8^ and 1.01 × 10^–8^ M. All these observations confirmed the critical effect of puerarin on inhibiting PTEN to ameliorate myocardial injury post-MI (Fig. 9J). To elucidate puerarin’s protective mechanisms in TBHP-induced apoptosis in cardiomyocytes, Western blotting and JC-1 staining were conducted, which showed that puerarin ameliorated the oxidative stress-induced mitochondrial injury and inhibited cardiomyocyte apoptosis through inhibiting the PTEN/AKT/GSK3β pathway (Fig. 9K–T, *p* < *0.01*).

## Discussion

**Fig. 10:**
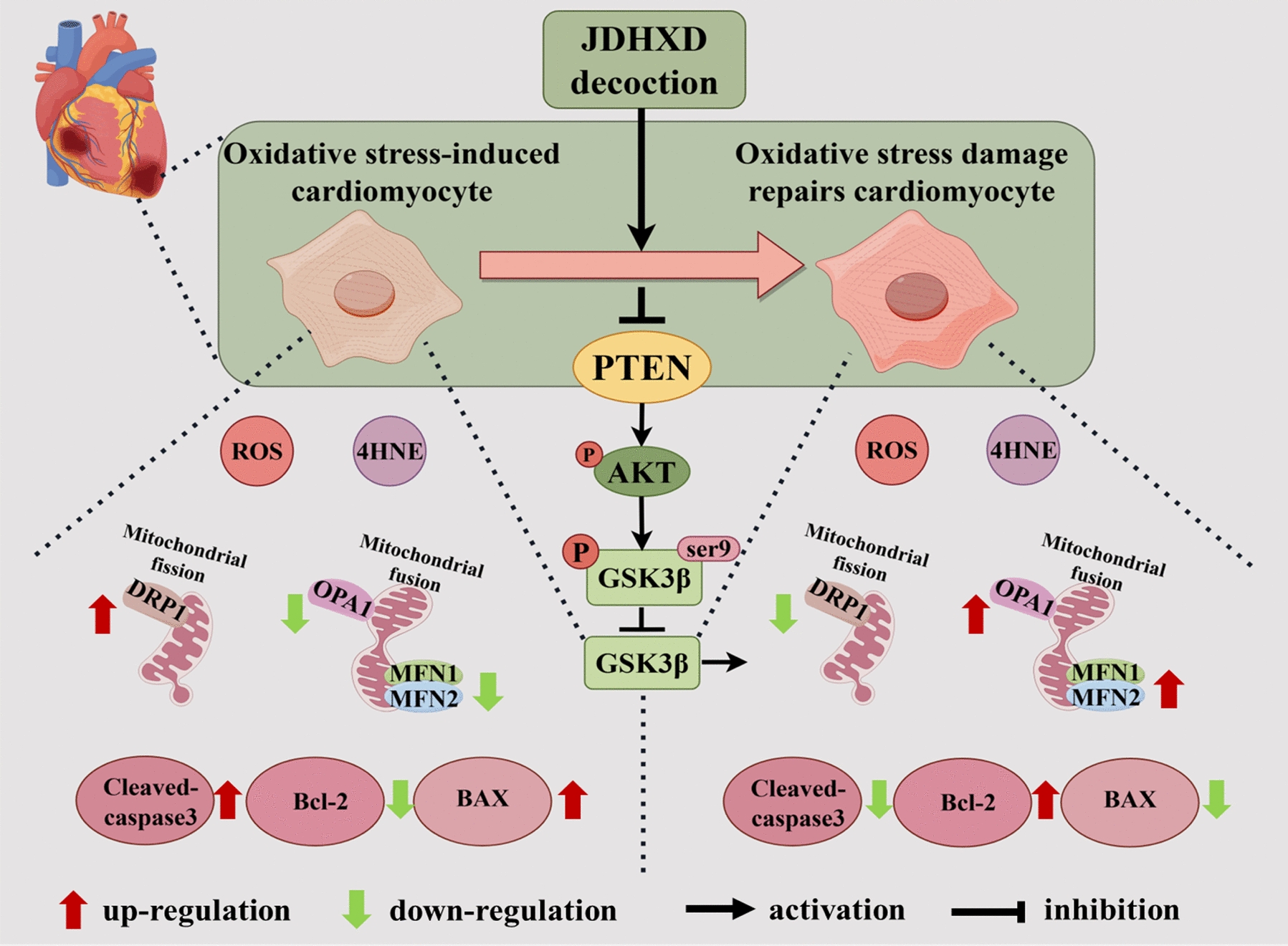
Schematic diagram illustrating the protective mechanism of JDHXD against MI. JDHXD improves oxidative stress-induced abnormal mitochondrial fission and fusion to suppress cardiomyocyte apoptosis partially through the PTEN/AKT/GSK3β signaling pathway after MI

MI constitutes a critical global health burden caused by reduced coronary blood flow. Epidemiological studies have reported the persistently high HF rates following MI during recent decades [34]. As documented, MI initiates a destructive pathological cascade marked by severe oxidative stress, profound mitochondrial dysfunction, as well as subsequent apoptosis of cardiomyocytes [5–7]. These above-mentioned events collectively promote unfavorable cardiac remodeling and accelerate HF progression. Although therapeutic approaches continually evolve, targeted strategies to preserve mitochondrial integrity and prevent post-MI cardiomyocyte apoptosis remain an urgent unmet clinical need [35, 36]. In this work, JDHXD conferred substantial cardioprotection post-MI by alleviating these key pathological processes. Most significantly, we revealed a core mechanistic pathway: JDHXD specifically suppressed oxidative stress-induced cardiomyocyte apoptosis in MI through alleviating mitochondrial injury. Both network pharmacology and transcriptomic results suggest its protective effect was achieved through suppressing PTEN while activating PI3K/AKT pathway, thereby inactivating the downstream GSK3β pathway and restoring the balance between mitochondrial fission/fusion post-MI (Fig. 10).

TCM offers substantial promise in cardiovascular therapeutics. According to relevant research, bioactive constituents of the traditional JDHXD formula provide distinct protection against ischemic damage [37–39]. From our findings, rigorous in vivo validation confirmed JDHXD's therapeutic effects: echocardiographic and histopathological assessment revealed that the key cardiac function indicators in MI mice improved significantly after JDHXD treatment. TUNEL/CTNT staining and WB suggest that the effect of JDHXD in improving myocardial injury after MI may be achieved by significantly inhibiting the apoptosis of cardiomyocytes after MI. Crucially, JDHXD conferred cardioprotection comparable to captopril, a reference ACE inhibitor used clinically for post-infarction management [40]. Since preserving cardiomyocyte viability is a fundamental therapeutic objective for limiting infarct progression and pathological remodeling [41], JDHXD's anti-apoptotic activity likely underpins its functional benefits.

As key cellular "powerhouses", mitochondria generate most ATP fueling biological processes in eukaryotes [42, 43]. Notably, the energetically demanding heart critically depends on mitochondrial ATP replenishment to sustain its contractile function [44–46]. Mitochondrial dysfunction, characterized by bioenergetic failure, constitutes a hallmark event in cardiac diseases including MI [47]. In ischemic myocardial injury, mitochondria act as both primary generators of destructive ROS and their principal targets [48]. This study confirmed that ROS accumulation and increased 4HNE (a lipid peroxidation biomarker) levels occurred after MI, and that oxidative stress damage led to mitochondrial ultrastructural damage, which specifically manifested as mitochondrial fragmentation and reduced surface area. Normal mitochondrial morphology is crucial for maintaining mitochondrial function and energy production [49, 50]. Typically, mitochondrial morphology is maintained via regulating mitochondrial fission/fusion via specific factors like OPA1, MFN1, and MFN2 (fusion proteins), and DRP1 (fission protein) [51, 52]. The mitochondrial fission/fusion imbalance induced by oxidative stress post-MI may result in secretion of Cytc as well as additional pro-apoptotic factors into cytoplasm and inducing cardiomyocyte apoptosis. As confirmed in this study, JDHXD improved oxidative stress-induced myocardial mitochondrial injury post-MI by maintaining the mitochondrial fission/fusion balance.

GSK-3β critically regulates mitochondrial activity, homeostasis, and function through multiple mechanisms [53]. Its activity is bidirectionally modulated by phosphorylation at Ser9 and Tyr216 residues (inactivating and activating GSK-3β separately) [27]. Evidence indicates that suppressing GSK-3β activity via enhanced inhibitory phosphorylation can promote cellular resistance to apoptosis [54]. Earlier ischemia–reperfusion models reveal GSK-3β activation within mitochondria as well as the physical interaction with mPTP complex [55]. Moreover, activated phospho-GSK-3β(Ser9) in mitochondria confers cardioprotection from ischemia–reperfusion injury in ischemic episodes [56]. We demonstrated that MI or TBHP stimulation induced GSK3β activation, provoking maladaptive alterations in mitochondrial dynamics proteins. Conversely, JDHXD treatment preserved mitochondrial integrity and attenuated cardiomyocyte apoptosis. Notably, GSK-3β activation modulated MMP via the phosphorylation of voltage-dependent anion channels, thereby enhancing cellular vulnerability to apoptosis [57]. In vitro studies verified that JDHXD suppressed GSK3β expression (elevated p-GSK3β(Ser9)), consequently reversing the TBHP-induced MMP dissipation and mitochondrial ROS overproduction. Collectively, GSK-3β is a key target through which JDHXD restores mitochondrial dynamics and function in MI. The candidate targets of JDHXD in treating MI were further investigated using network pharmacology and transcriptomics. The results indicated that JDHXD ameliorated myocardial injury post-MI via regulating PI3K/AKT pathway. GSK-3β serves as the Akt target, with phosphorylated Akt inactivating GSK-3β through phosphorylation of the Ser9 residue [58]. Our prior work establishes that PTEN suppression is essential for activating PI3K/AKT pathway attenuating cardiomyocyte apoptosis [20]. Recent studies have demonstrated that excessive activation of PTEN and its related signaling exacerbates mitochondrial dysfunction, mitophagy disorder, and dynamic imbalance in ischemic cardiomyocytes [59–61]. According to the present work, DEGs in JDHXD and PI3K/AKT signaling pathways were ranked, and PTEN was significantly enriched, suggesting that JDHXD may exert its function by inhibiting PTEN. Moreover, the results of Western blotting and IHC indicated that JDHXD significantly inhibited the phosphorylation expression of PTEN post-MI. Collectively, these findings suggest that PTEN/AKT/GSK3β signaling pathway serves as one important mechanism by which JDHXD ameliorates oxidative stress-induced mitochondrial injury and thereby inhibits cardiomyocyte apoptosis post-MI.

We further confirmed in vitro whether JDHXD exerted an anti-myocardial injury effect post-MI in association with PTEN by using a PTEN-selective inhibitor (Bpv) and constructing PTEN-overexpressing adenoviruses. The key experiment revealed that pharmacological PTEN inhibition using Bpv failed to alter the regulatory effects of JDHXD on cell viability, apoptotic markers (Cleaved-Caspase3, BAX, Bcl-2), oxidative stress parameters (4-HNE, ROS), mitochondrial fission/fusion proteins, or the expression status of PTEN/GSK3β pathway proteins themselves. This compellingly suggests that JDHXD achieves near-maximal inhibition of the relevant PTEN-mediated detrimental signaling. Conversely, adenoviral overexpression of PTEN in cardiomyocytes significantly attenuated the protective capacity of JDHXD. Specifically, PTEN overexpression partially nullified JDHXD's anti-apoptotic effects, weakened its attenuation of oxidative stress, impaired its rescue of MMP depolarization, and reduced its efficacy in inhibiting GSK3β activation and mitochondrial dynamics protein levels. Collectively, these results indicate that the role of JDHXD in ameliorating myocardial mitochondrial injury induced by oxidative stress and thereby inhibiting cardiomyocyte apoptosis is partially mediated by the PTEN signaling pathway.

TCM prescriptions display characteristics including complex effective components and diverse action targets. Therefore, it is the key to exploring specific effective components of JDHXD that regulate PTEN to exert its anti-myocardial injury effect post-MI. In this study, the specific components of JDHXD were investigated by UHPLC/Orbitrap-MS, including hydroxysafflor yellow A, puerarin, naringin, and kaempferol. Molecular docking, SPRi and experiments in vitro confirmed that puerarin was one of the main components of JDHXD that inhibited PTEN and improved myocardial injury post-MI. Several limitations of this study should be acknowledged. First, the present study demonstrated that PTEN signaling is involved in the protective effects of JDHXD against MI based on pharmacological inhibition, phenotypic rescue, and correlation analysis. However, direct causal evidence using PTEN knockout mice was not provided, which precludes definitive confirmation that PTEN is the sole or primary mediator. Second, the detailed crosstalk between PTEN and other mitochondrial regulatory pathways requires further investigation. Future studies using gene knockout or cardiac-specific conditional knockout models will help clarify the precise regulatory role of PTEN in the cardioprotective mechanism of JDHXD.

Collectively, JDHXD inhibits excessive mitochondrial fission and promotes mitochondrial fusion induced by oxidative stress partially through suppressing the PTEN/AKT/GSK3β pathway, thereby attenuating cardiomyocyte apoptosis post-MI. Our results suggest that the PTEN/AKT/GSK3β axis serves as a key pathway underlying the protective effects of JDHXD against myocardial injury by inhibiting cardiomyocyte apoptosis, and highlight the potential of JDHXD as a promising adjunctive therapy for MI.

## Supplementary Information

Supplementary material 1.

## Data Availability

The data produced from this study are available from the corresponding author on reasonable request.

## Abbreviations

ANOVA: Analysis of variance
Bpv: Bisperoxovanadium
cTNT: Cardiac Troponin T
CFs: Cardiac fibroblasts
DMEM: Dulbecco’s modified eagle medium
FBS: Fetal bovine serum
GSK-3β: Glycogen synthase kinase 3β
HF: Heart failure
HNE: Hydroxynonenal
HE: Hematoxylin–eosin
IHC: Immunohistochemistry
IOD: Integral optical density
JDHXD: Jie Du Huo Xue Decoction
LAD: Left anterior descending
LVDs: Left ventricular end-systolic diameter
LVDd: Left ventricular end-diastolic dimension
LVFS: Left ventricular fractional shortening
LVEF: Left ventricular ejection fraction
MI: Myocardial infarction
mPTP: Mitochondrial permeability transition pore
MOI: Multiplicity of infection
MMP: Mitochondrial membrane potential
NCMs: Neonatal rat cardiomyocytes
PTEN: Phosphatase and tensin homolog
rAV-PTEN: Recombinant adenovirus encoding mouse PTEN
rAV-GFP: Recombinant adenovirus-green fluorescent protein
ROS: Reactive oxygen species
TCM: Traditional Chinese medicine
TBHP: Tert-butyl hydroperoxide
TEM: Transmission electron microscopy
UHPLC: Ultra-high performance liquid chromatography
