# Supplementary material for: Jiedu Huoxue decoction inhibits cardiomyocyte apoptosis via PTEN/AKT/GSK3β-mediated mitochondrial dynamics in myocardial infarction: an integrative study of network pharmacology, transcriptomics and molecular docking
Source: Chin Med. 2026 Jun 29;21:176. doi: 10.1186/s13020-026-01444-7 (PMC13312624; doi:10.1186/s13020-026-01444-7)
Supplement: Supplementary file 1 — Supplementary material 1. [file 13020_2026_1444_MOESM1_ESM.docx]

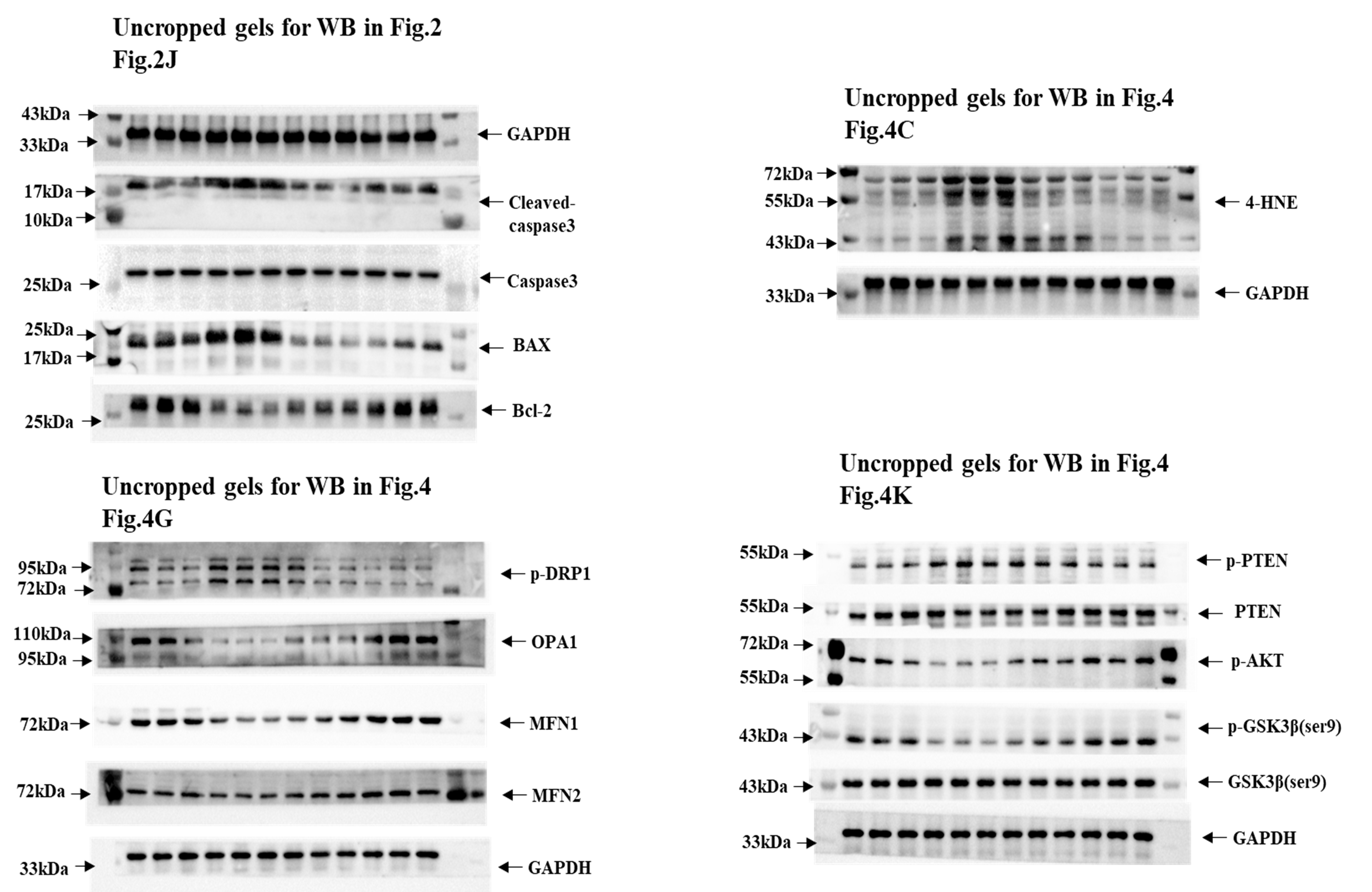


**Fig. S1 All the blot results in the manuscript’s Figures 2 and 4 are presented with uncropped and minimally adjusted images.**

**
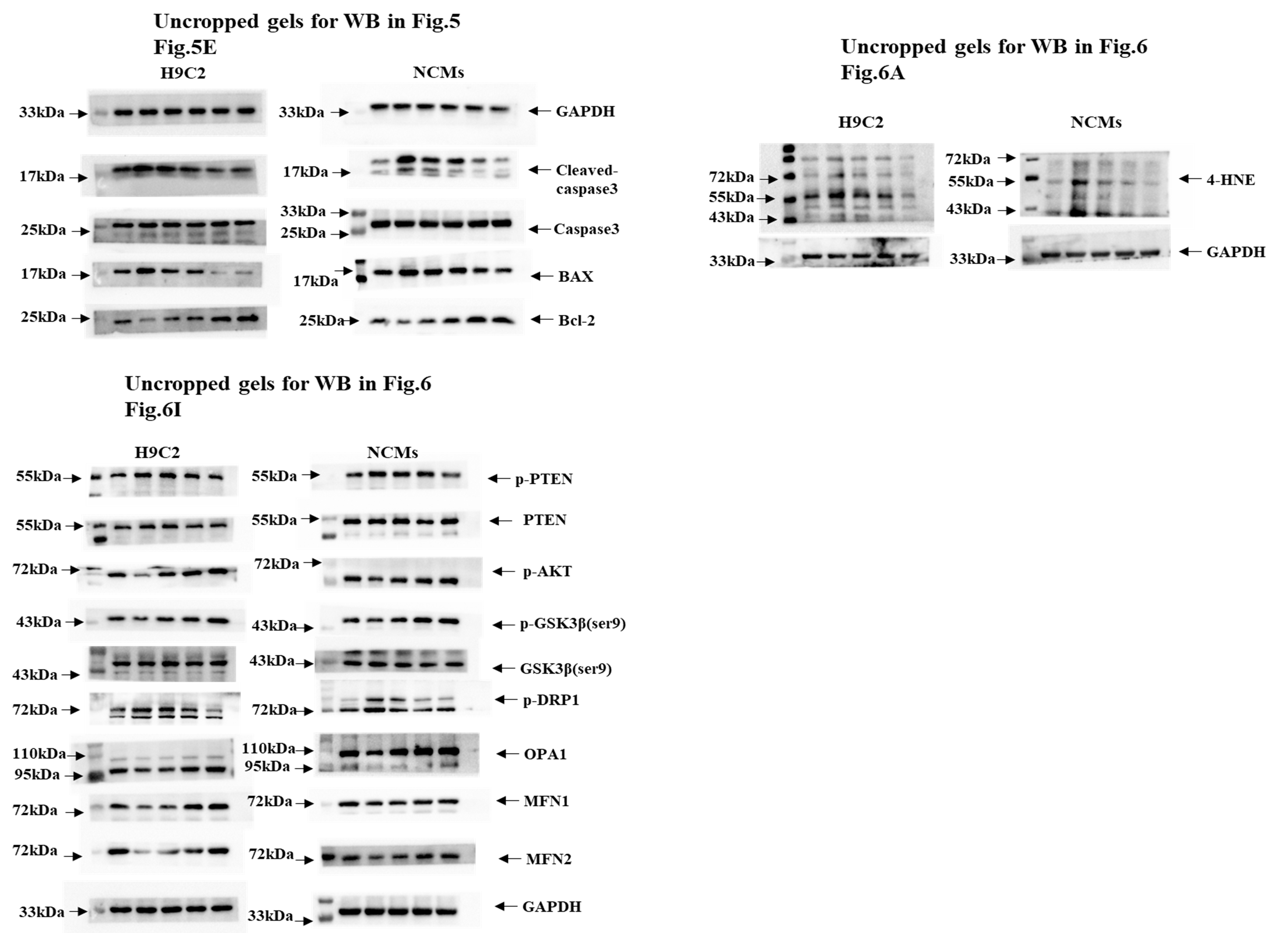
**

**Fig. S2 All the blot results in the manuscript’s Figures 5 and 6 are presented with uncropped and minimally adjusted images.**

**
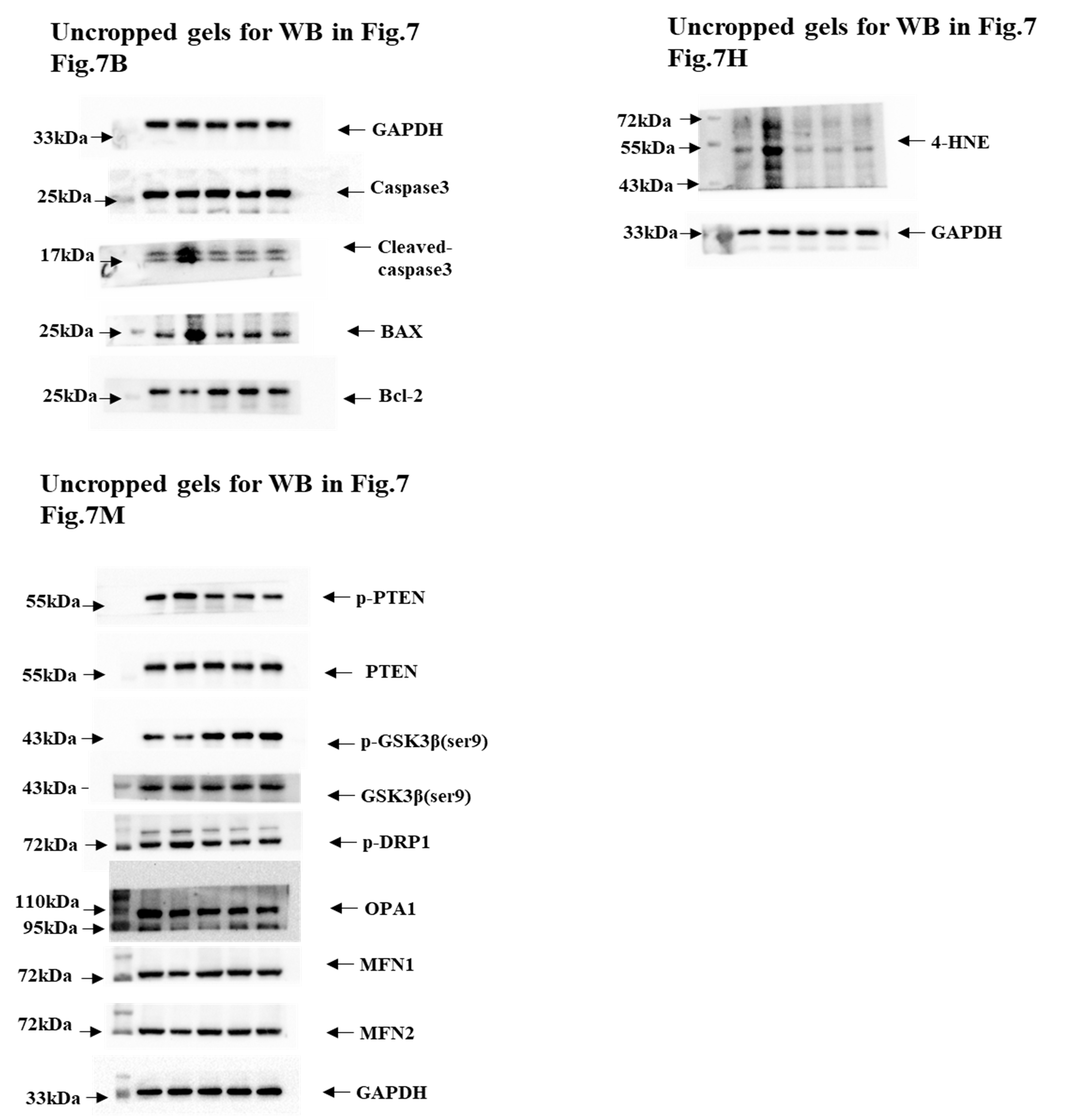
**

**Fig. S3 All the blot results in the manuscript’s Figure 7 are presented with uncropped and minimally adjusted images.**

**
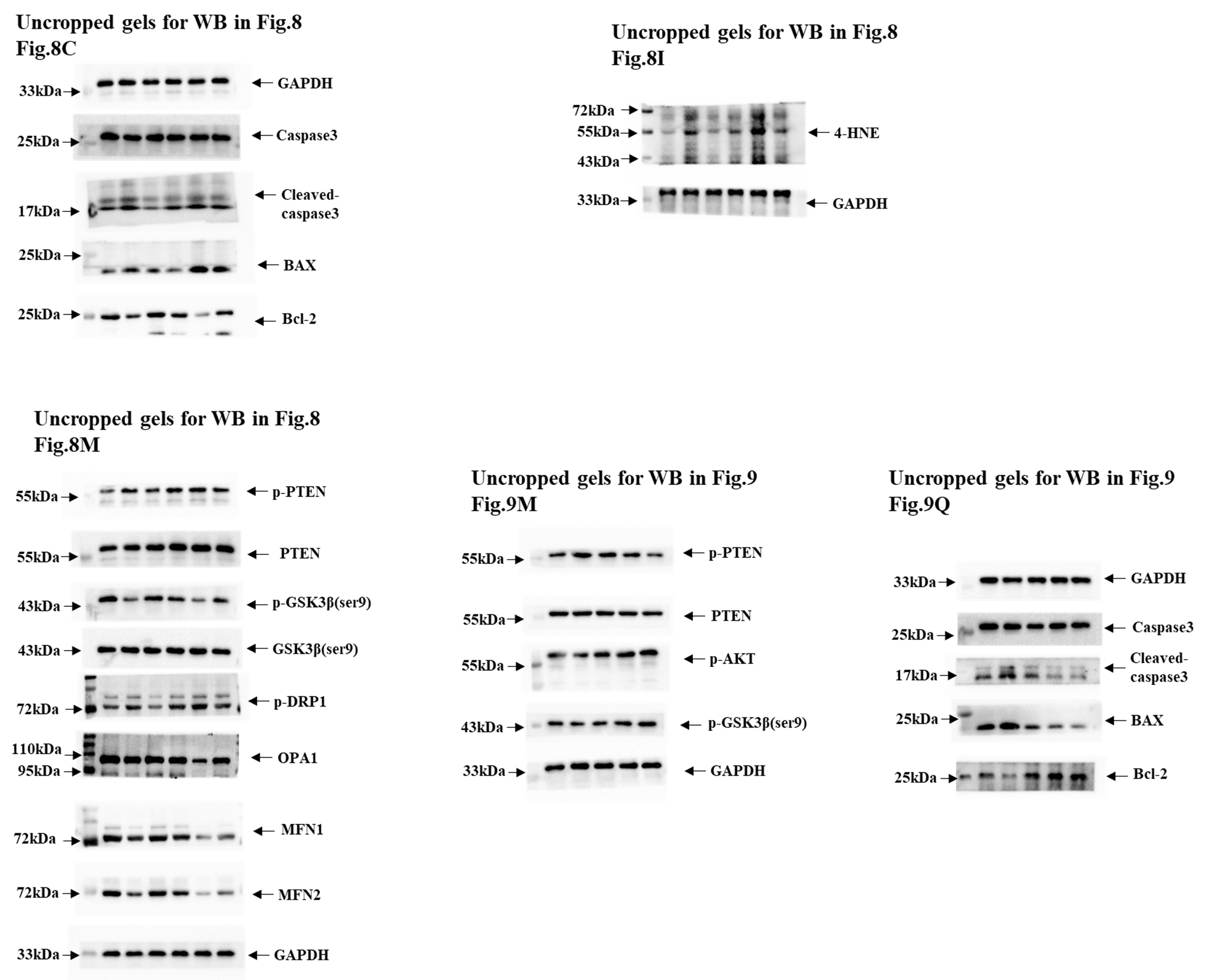
**

**Fig. S4 All the blot results in the manuscript’s Figures 8 and 9 are presented with uncropped and minimally adjusted images.**
